# Subacute Changes in Cleavage Processing of Amyloid Precursor Protein and Tau following Penetrating Traumatic Brain Injury

**DOI:** 10.1371/journal.pone.0158576

**Published:** 2016-07-18

**Authors:** Casandra M. Cartagena, Andrea Mountney, Hye Hwang, Adam Swiercz, Zoe Rammelkamp, Angela M. Boutte, Deborah A. Shear, Frank C. Tortella, Kara E. Schmid

**Affiliations:** Brain Trauma Neuroprotection and Neurorestoration Branch, Center for Military Psychiatry and Neuroscience, Walter Reed Army Institute of Research, Silver Spring, Maryland, United States of America; University of S. Florida College of Medicine, UNITED STATES

## Abstract

Traumatic brain injury (TBI) is an established risk factor for the development of Alzheimer’s disease (AD). Here the effects of severe penetrating TBI on APP and tau cleavage processing were investigated in a rodent model of penetrating ballistic-like brain injury (PBBI). PBBI was induced by stereotactically inserting a perforated steel probe through the right frontal cortex of the anesthetized rat and rapidly inflating/deflating the probe’s elastic tubing into an elliptical shaped balloon to 10% of total rat brain volume causing temporary cavitation injury. Separate animals underwent probe injury (PrI) alone without balloon inflation. Shams underwent craniectomy. Brain tissue was collected acutely (4h, 24h, 3d) and subacutely (7d) post-injury and analyzed by immunoblot for full length APP (APP-FL) and APP beta c-terminal fragments (βCTFs), full length tau (tau-FL) and tau truncation fragments and at 7d for cytotoxic Beta amyloid (Aβ) peptides Aβ40 and Aβ42 analysis. APP-FL was significantly decreased at 3d and 7d following PBBI whereas APP βCTFs were significantly elevated by 4h post-injury and remained elevated through 7d post-injury. Effects on βCTFs were mirrored with PrI, albeit to a lesser extent. Aβ40 and Aβ42 were significantly elevated at 7d following PBBI and PrI. Tau-FL decreased substantially 3d and 7d post-PBBI and PrI. Importantly, a 22 kDa tau fragment (tau22), similar to that found in AD, was significantly elevated by 4h and remained elevated through 7d post-injury. Thus both APP and tau cleavage was dramatically altered in the acute and subacute periods post-injury. As cleavage of these proteins has also been implicated in AD, TBI pathology shown here may set the stage for the later development of AD or other tauopathies.

## Background

Amyloid plaques and tau aggregation into neurofibrillary tangles are the two hallmarks of Alzheimer’s disease (AD) [1]. The pathogenic cleavage processing of amyloid precursor protein (APP) is thought to be one of the starting blocks leading ultimately to these hallmark characteristics of AD and involves increased β-secretase cleavage leading to the production of beta amyloid (Aβ) fragments 40 and 42 (Aβ40, Aβ42) and intracellular C-terminal fragments (βCTFs) [2]. Likewise, both the pathogenic cleavage and phosphorylation of tau are thought to contribute to the long-term development of tau tangles, and although this process is less characterized, recent studies indicate tau cleavage can function similar to prions and accelerate tau aggregation [3–5]. The study of AD genetic pre-dispositions has shown that individuals possessing certain familial mutations may develop AD early in life (≥30 yrs) [6]. However, for individuals lacking any specific familial mutation traumatic brain injury (TBI) is considered one of the strongest risk factors for the development of late-onset, sporadic AD. Converging data from a number of clinical studies have shown head trauma suffered earlier in life leads to increased rates in the subsequent development of sporadic Alzheimer’s disease pathology [7–13]. These studies suggest that the pathology triggered by TBI may contribute to long-term mechanisms altering the processing of key AD markers APP and tau in the absence of any known AD familial mutations. Clinical studies of human biomarkers have also shown acute changes in APP and tau cleavage processing following TBI [14–16]. In transgenic animal studies overexpressing genes involved in familial AD, TBI has been shown to increase beta-cleavage of APP and tau cleavage/aggregation following brain injury [17–23]. However, with few exceptions in mouse [24, 25], such molecular studies have almost exclusively been conducted using transgenic animals and over-expression systems which may not accurately parallel the TBI-induced molecular changes potentially related to late-onset sporadic AD. Here the effects of severe penetrating ballistic-like brain injury (PBBI), as well as probe injury (PrI), on APP and tau cleavage processing were investigated in the acute (4, 24h, 3d) to sub-acute periods (7) post-TBI using a non-transgenic rat model.

## Materials and Methods

### Animals

Male Sprague-Dawley rats were used in these experiments. All surgical procedures were performed under anesthesia (isoflurane). Prior to euthanasia rats were deeply anesthetized with ketamine/xylazine and euthanasia performed by transcardial exsanguination and then decapitation. Postoperative analgesics were not used following brain injury due to potentially conflicting interactions of analgesics on the endpoints being measured. Both narcotic and non-narcotic analgesics, as well as anti-inflammatory treatments, possess the potential for drug induced neuroprotection, which would severely compromise the proposed studies. Routine postoperative administration of analgesics is thus contraindicated by virtue of their established neuroprotection efficacy and their negative impact on the experimental design and data interpretation. Therefore, the animals were monitored to determine when early humane endpoints were needed. Animals displaying signs of pain and distress including but not limited to decreased activity, excessive licking/scratching, self-mutilation, abnormal locomotion such as stumbling, falling, or writhing, unusual aggressiveness, hiding, rough/stained hair coat, abnormal stance or arched back, porphyrin staining, rapid/shallow breathing, decreased appetite, or tremors would then be evaluated by a Clinical Veterinarian to determine if these are typical signs of the brain injury model or if euthanasia is warranted as an early humane endpoint. However, no animals showed signs of distress beyond that typical of the brain injury model and no animals died prior to the experimental endpoint. All procedures were approved by the Walter Reed Army Institute of Research (WRAIR) Institutional Animal Care and Use Committee (IACUC) and performed in facilities accredited by Association for Assessment and Accreditation of Laboratory Animal Care International (AAALACi). Research was conducted in compliance with the Animal Welfare Act and other federal statutes and regulations relating to animals and experiments involving animals and adheres to principles stated in the Guide for the Care and Use of Laboratory Animals, NRC Publication, 2011 edition.

### Penetrating ballistic-like brain injury

**Fig 1:**
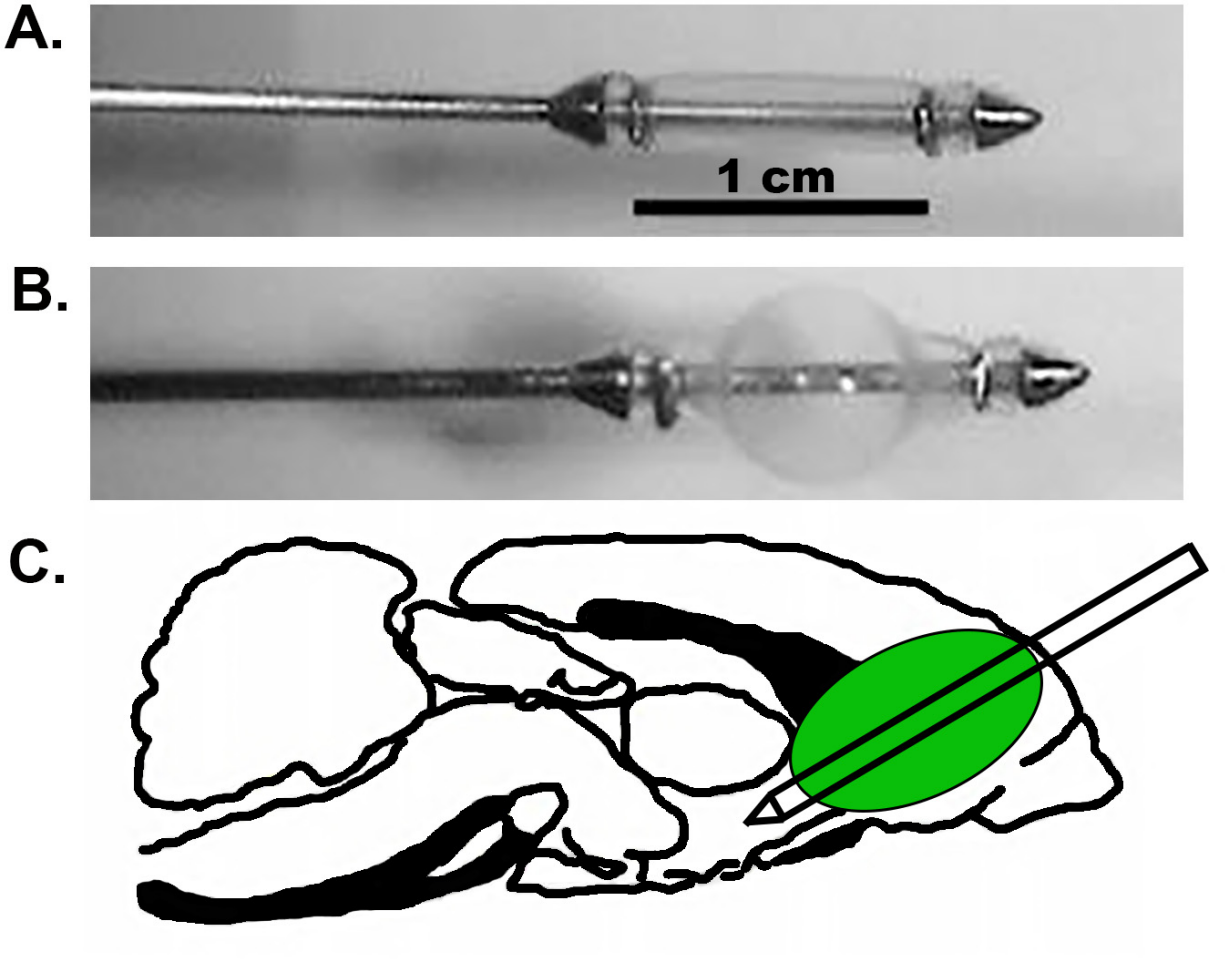
PBBI model (A) Visualization of PBBI probe (B) PBBI probe with balloon inflation (C) anatomical location of injury tract with probe alone and probe with balloon inflation.

Unilateral frontal PBBI was performed using a simulated injury device as previously described [26, 27]. The PBBI apparatus consists of a computer-controlled simulated ballistic injury device (Mitre Corp, McLean VA) attached to a specially designed PBBI probe made of a 20 G stainless steel tube with fixed perforations along one end sealed by airtight elastic tubing. Animals were anesthetized with 2% isoflurane and positioned in a stereotaxic frame. A craniectomy was performed and the probe was manually inserted through the cranial window through the right frontal pole of the brain (+4.5mm antero-posterior, +2mm medio-lateral from bregma) at an angle of 50° from the vertical axis and 25° counter-clockwise from the anterior-posterior axis to a distance of 1.2 cm from the dura surface of the brain. Following this, the computer-controlled pulse generator was activated to rapidly inflate/deflate (i.e. <40 msec) the elastic tubing on the probe to an elliptical-shaped balloon to a size equal to ~10% of the total brain volume mimicking a simulated shock-wave thereby causing the temporary cavity and subsequent brain lesion. In addition, separate animals received an injury induced by probe insertion alone (PrI). Sham control animals received all surgical procedures except for probe insertion. A visualization of the probe, probe with balloon inflation, and anatomical location of the injury tract can be seen in Fig 1.

### Tissue Collection

Fresh whole brains were placed in an adult rat brain slice matrix block (Braintree Scientific, Braintree, MA) and an ipsilateral coronal slice starting 3 mm from the frontal pole and ending 5 mm from the frontal pole (2 mm wide, bregma -1.8mm to bregma 0.2mm) was collected, snap frozen in super cooled isopentane for later analysis by western blots. Samples were collected at 4h, 24h, 3d, and 7d post-injury (sham, PrI, and PBBI, n = 8–10 per group). An ipsilateral coronal slice starting 5 mm from the frontal pole and ending 7 mm from the frontal pole (2 mm wide, bregma 0.2mm to bregma +3.2mm) was collected 7 days post injury (sham, PrI, PBBI, n = 10/ group), snap frozen in super cooled isopentane for later analysis by ELISA. The ipsilateral tissue slice contained both the ipsilateral core and perilesional injury zone. Additional pilot studies were done with collecting dissected regions of interest (ROIs), ipsilateral frontal cortex, striatum, hippocampus, and residual midbrain, at 24h post-injury (sham, PBBI n = 4 per group) and 1m post-injury (sham, PrI, PBBI n = 3 per group).

### APP and tau Positive Controls

Lysate from mouse N2A cells overexpressing huAPP (wildtype) (provided by Dr. Mark Burns, Georgetown University, Washington DC) were used as a positive control for APP-FL expression. In addition whole brain lysate from a 3 month old 3xTg-AD transgenic mouse, strain B6; 129-Psen1^tm1Mpm^ Tg (APPSwe, tauP301L)1Lfa/J (Jackson Laboratory, Bar Harbor, ME) was used as a positive control for APP and tau expression and related cleavage products.

### Western Blotting

**Fig 2:**
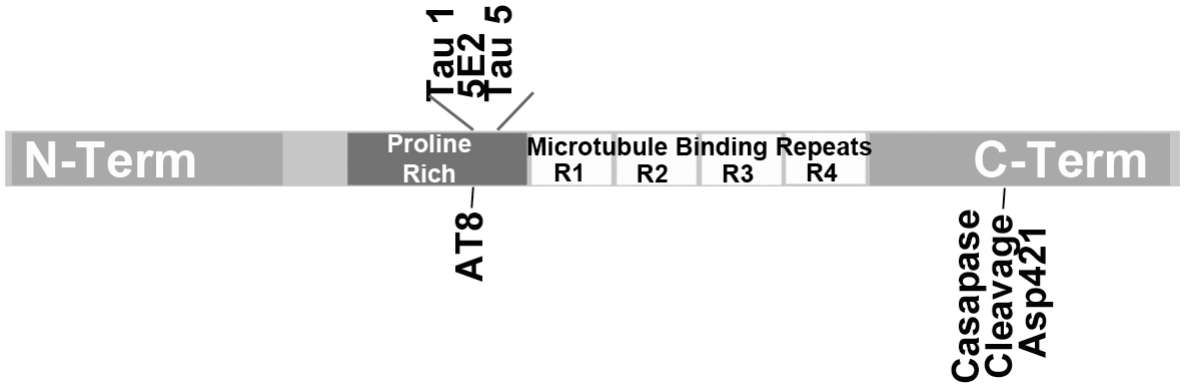
Domain location of antibodies Tau1, 5E2, Tau5 and caspase cleavage at Asp421.

Tissue was homogenized on ice in 1XRIPA buffer with Halt Protease inhibitors (Thermo Scientific / Pierce, Waltham, MA). Protein loaded for each sample was normalized based on BCA assay total protein concentration and equal total protein was loaded for all wells. Each lane represents a separate rat. Blots were blocked in 5% milk and 1XPBS, pH 7.4. For APP-FL and APP-CTF detection membranes were cut at the 25 kDa marker. The upper portion was probed with rabbit anti-APP primary antibody (recognizing C-terminal epitope KMQQNGYENPTYKFFEQMQN of APP, Sigma A8717, St. Louis, MO) at 1:1000 in block solution. The lower portion was probed with anti-APP antibody at 1:500. For tau and tau cleavage product detection blots were probed with mouse anti-tau (Clone 5E2, recognizing proline rich domain epitope SLPTPPTREPKKVAVVRTPP of total tau [28], Millipore 05–348, Billerica, MA) at 1:500 in block. Blots were washed 3 times with 1XPBS containing 0.01% tween-20 and incubated with secondary antibody (1:20,000, anti-mouse IgG GE Healthcare NA9310V or 1:5000, anti-rabbit IgG NA9340V, Wauwatosa, WI). Signal was detected with Clarity Western ECL. Blots were re-probed for beta actin. Band intensity was analyzed using an LAS4000 with ImageQuant TL software (GE Healthcare, Pittsburgh, PA). Tau cleavage products were further evaluated using additional anti-tau antibodies [Tau1 (recognizing proline rich domain epitope PKSGDRSGTSSPGSPGTPG of total tau [28]) (1:2000, Millipore MAB3420, Billerica, MA); Tau5 (recognizing proline rich domain epitope PPTREPKK of total tau [29]) (1:1000, Abcam ab80579, Cambridge, MA); tau truncated at Asp421 when cleaved by caspases (Caspase 3, 7, or 8), epitope CSSTGSIDMVD (1:1000, Invitrogen AHB0061, Camarillo, CA; AT8 (tau phosphorylation at S202 and Thr205, epitope PGpSPGpTPG [29]) (1:1000, Cell Signaling Technology 11834, Danvers, MA)]. Epitope binding of tau antibodies are displayed in relation to tau domains in Fig 2.

### Chemiluminescent Aβ ELISAs

Tissue was homogenized on ice using a Dounce homogenizer in a 1XTBS solution with protease inhibitors (Halt 1:100) and 2mM EDTA (1ml). Triton X-100 (1% total volume) was added to homogenate prior to centrifugation (1:100). Samples were spun in an ultracentrifuge (Optima L-100 XP, Beckman Coulter) at 165,000xg for 40 minutes at 4°C. Supernatant was removed for use with ELISA assay. Chemiluminescent ELISAs detecting Aβ40 (Covance SIG-38950) and Aβ42 (Covance SIG-38952) were used to measure relative amounts of Aβ in homogenized tissue samples in duplicate and manufacturer provided standards in triplicate. Standards were serially diluted in dilution buffer provided by the manufacture while samples were run without dilution (50μl/ well). The 96 well ELISA plate was aligned over a non-parallax (NP) tray and the optical density of each well was captured and analyzed with an LAS4000 docking station and ImageQuantTL software (GE Healthcare). Optical density measures for each sample were normalized using total protein levels (BCA assay).

### Statistical Analysis

Molecular measures at each time point were first analyzed for outliers using the ROUT method (Q = 0.1000%). Measures were then analyzed to Gaussian distribution using the D’Agnotino and Pearson omnibus normality test, the Shapiro-Wilk test, and the Kolmogorov-Smirnov test. Several data sets failed to show a Gaussian distribution and logarithmic conversion did not lead to Gaussian distribution. Therefore each time point was analyzed using the non-parametric Kruskal-Wallis (K-W) test. The appropriateness of two-way ANOVA across time is debatable as samples are normalized to controls at each time point. Furthermore this analysis assumes a Gaussian distribution as well as a linear relationship between dependent and non-dependent variables [30, 31], while molecular responses following TBI frequently fluctuate or have multi-phasic patterns. However, in the interest of being comprehensive, two way ANOVA analyses across time were conducted using the Holm-Sidak multiple comparison test in addition to (K-W) analysis at discrete time points. Changes were considered significant with p<0.05. All analyses were conducted using Prism (Graphpad, La Jolla, CA).

## Results

### Detection of APP-FL, βCTF, and αCTFs in the non-transgenic rat

**Fig 3:**
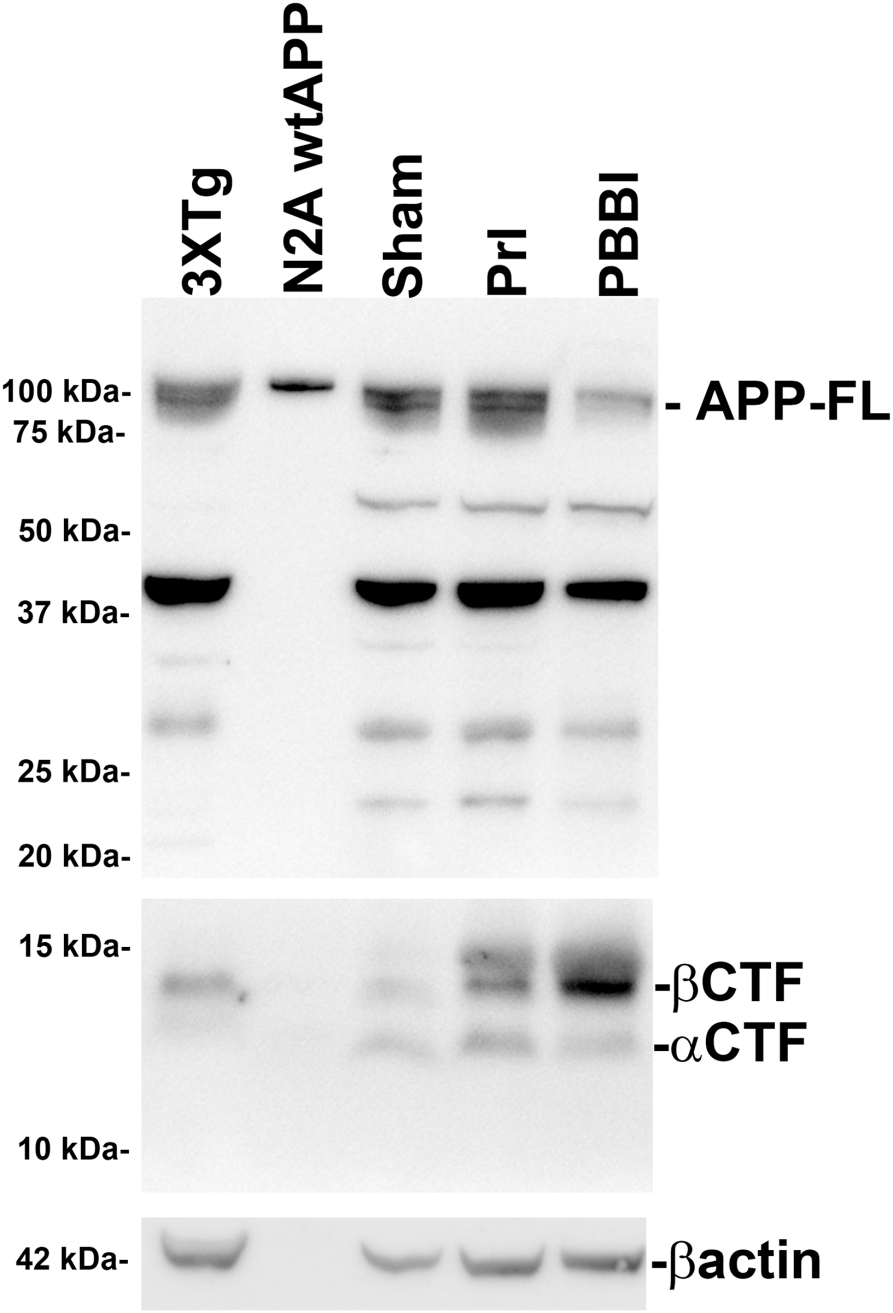
Detection of FL APP and cleavage products following penetrating TBI. Protein levels from sham, PrI and PBBI at 3d post-injury were evaluated for the detection of bands corresponding to APP-FL, βCTF, αCTF, and βactin and compared to positive controls including transgenic AD model mouse brain lysate (3XTg-AD) and cell culture lysate from N2A cells transfected with human APP (N2A wtAPP).

In our initial experiments, it was determined whether APP-FL (100 kDa), βCTFs (14 kDa), or the alternative α-secretase APP C-terminal fragment (αCTFs, 12 kDa) were detectable in the brain tissue of the non-transgenic rat using western blot and whether these factors are altered following either PrI or PBBI. Using brain tissue from sham, PrI, and PBBI 3d post-injury, it was found that APP-FL, βCTFs, and αCTFs, were detectable in the absence of a transgenic modification in the rat brain. In addition, it was found that APP-FL levels were clearly decreased following PBBI and that βCTFs were increased after both PrI and PBBI (Fig 3).

### Acute TBI induced changes in APP-FL, βCTF, αCTF, and Aβ

**Fig 4:**
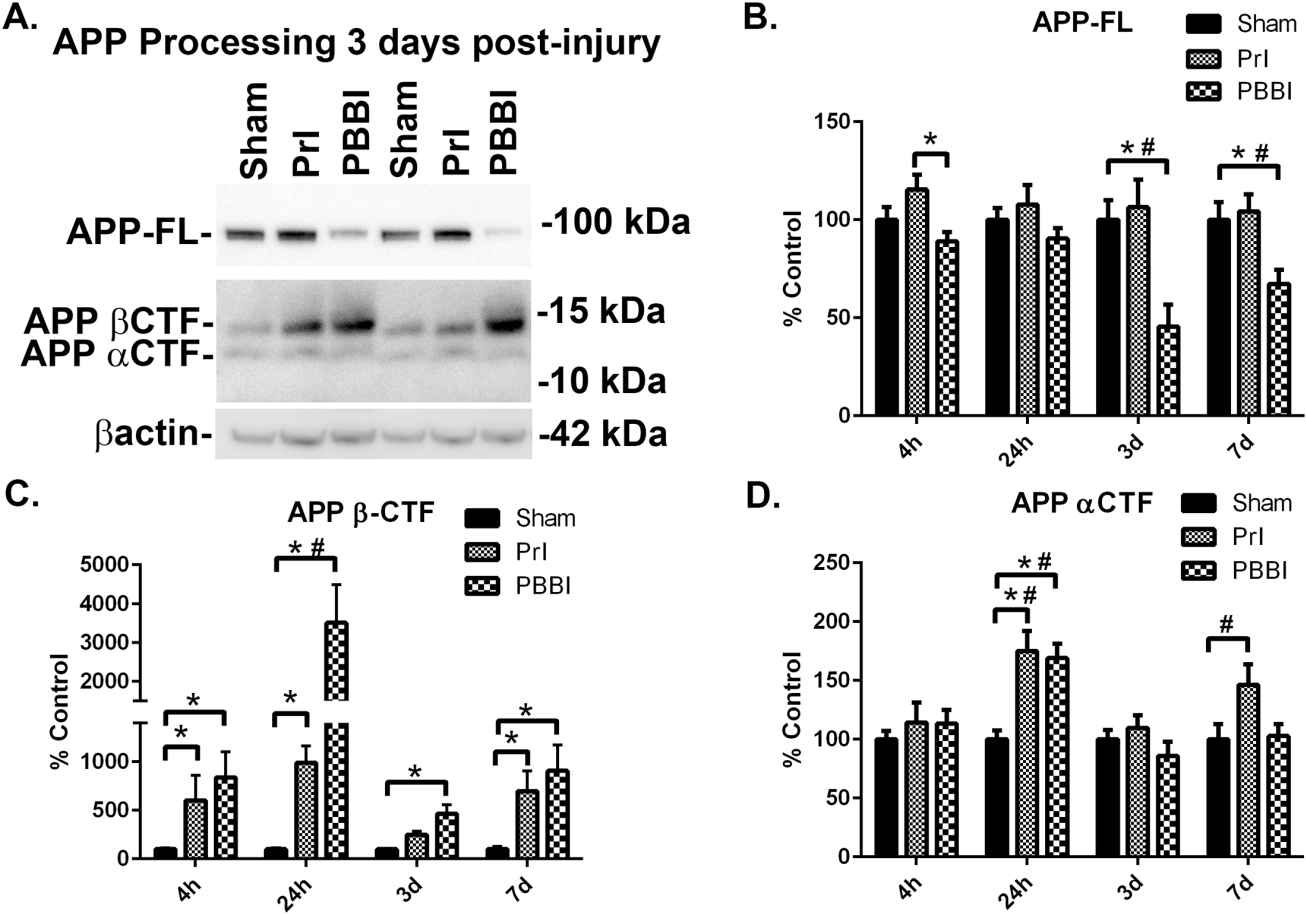
Loss of APP and accumulation of βCTFs after TBI. Protein levels from sham, PrI and PBBI were evaluated by Western blot at time points 4h, 24h, 3d and 7d days post injury. Bands were measured and normalized to corresponding βactin levels. A representative blot at 3d post injury is shown in (A). Quantification corresponding to (B) APP-FL, (C), APP βCTFs, and (D) APP αCTFs show significant loss of APP-FL with PBBI and significant increases in βCTFs at all time-points for PBBI and at 7d for PrI. APP αCTFs were significantly increased post-PrI and -PBBI at 24h and significantly decreased at 3d post-PBBI Statistical analysis of changes at each time point was conducted (K-W, * p<0.05, 2 way ANOVA # p<0.05; error bars: standard error of the mean, n = 8–10 per group).

Next, APP-FL, βCTF and αCTF levels were evaluated more comprehensively, over four time points spanning the acute to sub-acute period following injury (4h, 24h, 3d, and 7d). At 3 days post-injury, representative blot showed loss of APP-FL with PBBI and concomitant increases in βCTF following PrI and PBBI (Representative blot: Fig 4A, Additional blots for all time points: S1 through S4 Figs). β-actin levels were measured for each blot but not used for normalization purposes due to significant increases at later time points (3d, 7d) post-injury (S5 Fig) as has been shown in other neuropathology models [32]. Changes in βCTF levels did not follow a linear pattern but rather showed peak increases at 24h post-PBBI followed by more limited increases at later time points (3d, 7d). ROUT analysis did not identify any outlier measurements for APP-FL, βCTF and αCTF measurements. Analysis of quantification indicated that while APP-FL levels were stable at all time-points evaluated following PrI, PBBI led to significant loss of APP-FL at 3d (-55%, K-W and 2way ANOVA p<0.05) and 7d (-33%, K-W and 2 way ANOVA p<0.05) (Fig 4B). PBBI significantly increased βCTF levels at 4h (735%, K-W p<0.05), 24h (3407%, K-W and 2 way ANOVA p<0.05), 3d (365%, K-W p<0.05) and 7d (594%, K-W p<0.05). PrI led to a similar pattern of increased levels in βCTF but with lower amplitude which reached significance at 4h (501%, K-W p<0.05), 24h (891%, K-W p<0.05) and 7d (891%, K-W p<0.05) (Fig 4C). Both PrI and PBBI led to acutely increased αCTF levels 24h post injury (PrI: 75%,; PBBI: 69%, K-W p<0.05). These acute changes in αCTF levels resolved by 3d post-injury but showed an upward trend 7d following PrI injury (46%, 2wANOVA p<0.05) (Fig 4D).

**Fig 5:**
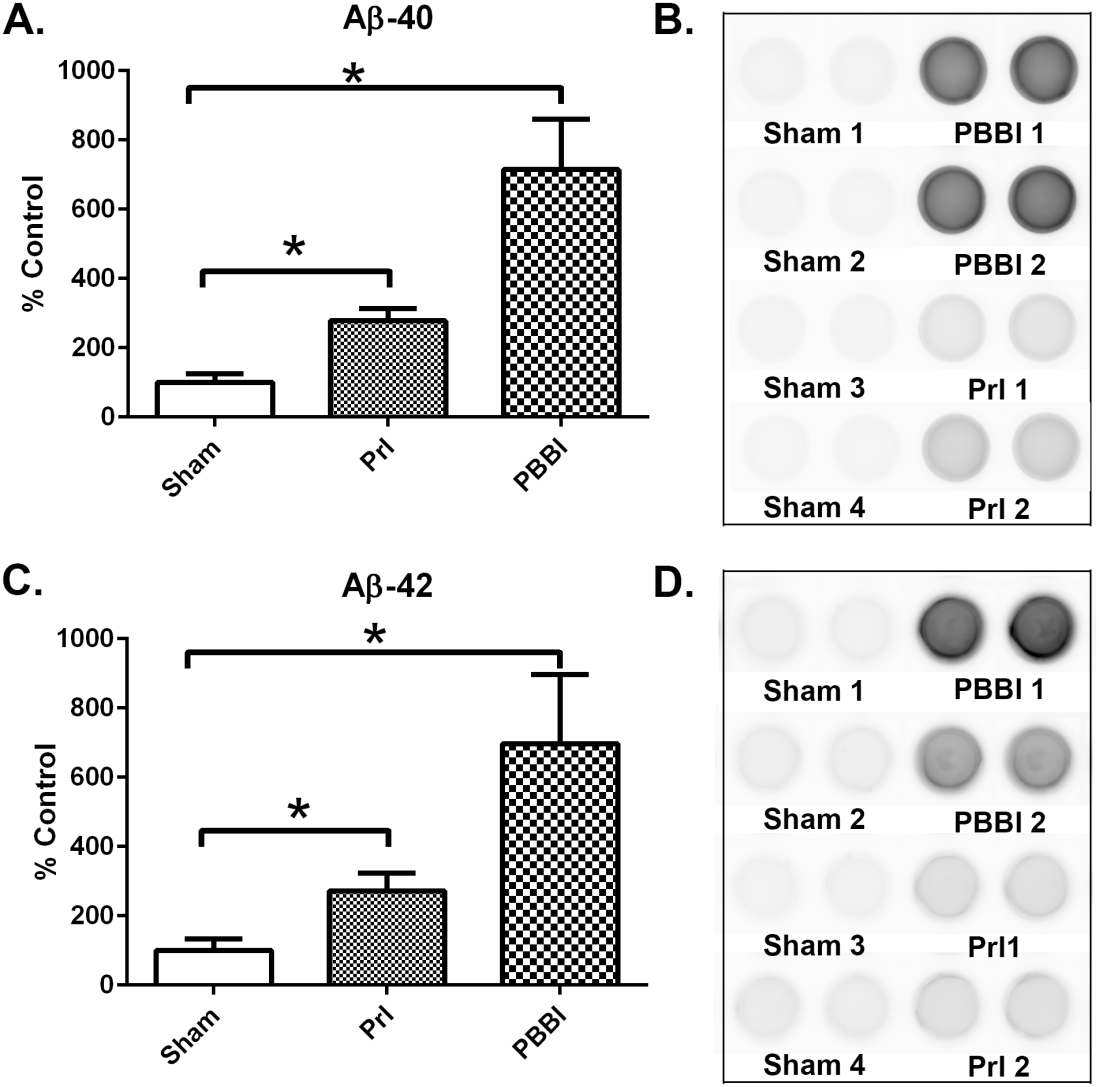
Increased Aβ40 and Aβ42 7 days post-TBI. Amyloid levels from Sham, PrI and PBBI were evaluated by chemiluminescent ELISAs 7d post-injury. (A) Quantification of Aβ40 (B) Representative visualizing of Aβ40 chemiluminescent ELISA wells. (C) Quantification of Aβ42 (D) Representative visualizing of Aβ42 chemiluminescent ELISA wells. Both Aβ40 and Aβ42 were significantly upregulated by PrI and PBBI 7d post-injury with PBBI inducing significantly more Aβ40 and Aβ42 than PrI. Statistical analysis of changes was conducted (K-W, * p<0.05; error bars: standard error of the mean, n = 10 per group).

Next, Aβ40 and Aβ42 levels were examined at in brain tissue following PrI and PBBI. A chemiluminescent based ELISAs specific for either Aβ40 or Aβ42 was used to evaluate Aβ40 and Aβ42 levels. Samples were assayed undiluted to maximize detection of low quantity amyloid in this non-transgenic model where amyloid was not artificially overexpressed. Standard curves and blanks including diluent buffer showed excessive background. However, undiluted samples produced highly consistent measurements across sample replicates and with clearly distinguishable differences across injury groups in comparison to sham (Representative wells: Fig 5B and 5D, All wells: S6 to S7 Figs). Consequently, Aβ levels were normalized to total protein levels and expressed semi-quantitatively as percent of control. ROUT analysis did not identify any outliers. Both PrI and PBBI increased the formation of Aβ40 (PrI: 178%,; PBBI: 615%, K-W, * p<0.05, Fig 5A) and Aβ42 (PrI: 172%,; PBBI: 597%, K-W, * p<0.05, Fig 5C) at 7 d post-injury.

### Acute TBI induced changes in tau-FL and tau cleavage

**Fig 6:**
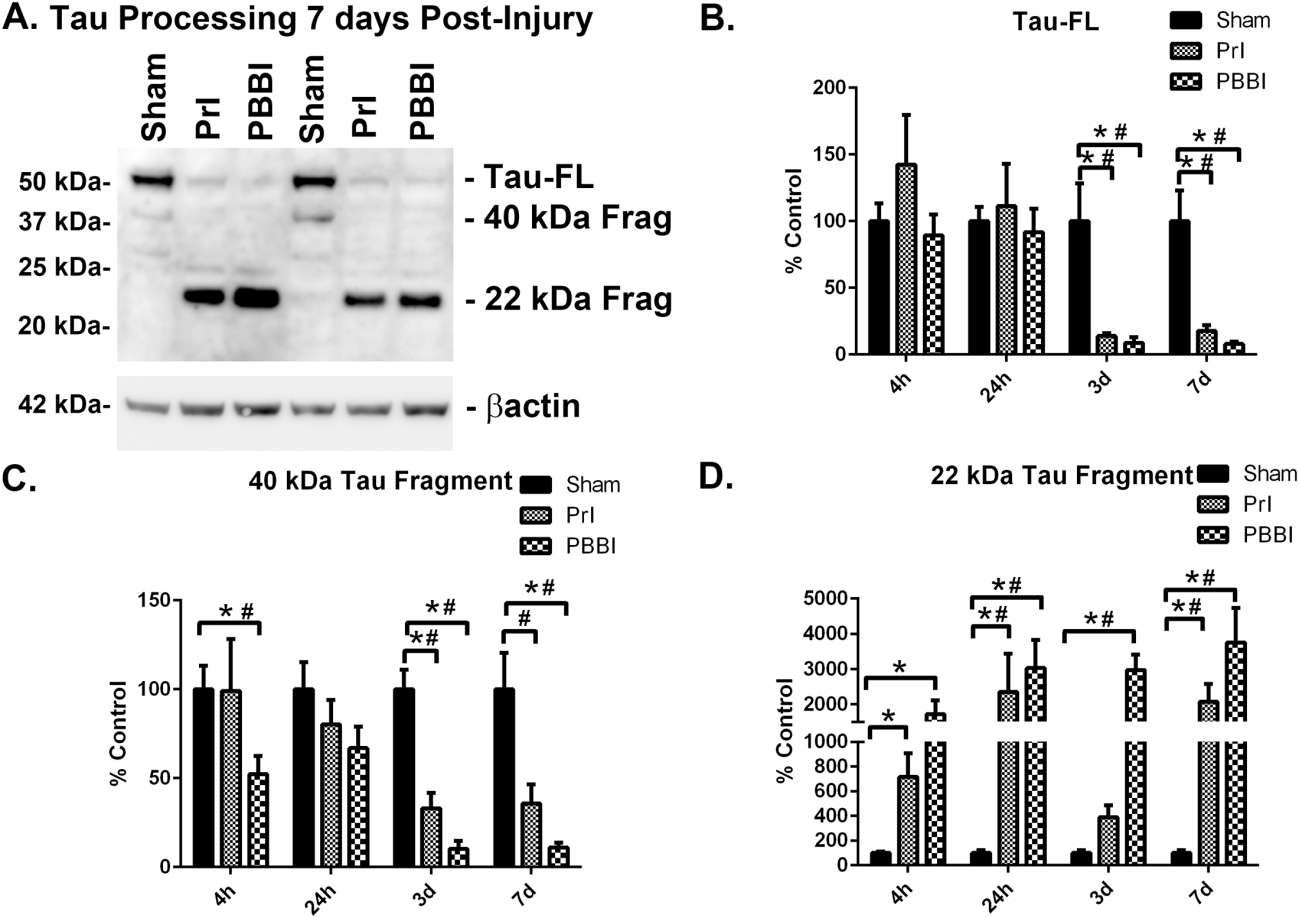
Detection of FL *tau* and *tau* fragments following penetrating TBI. Protein levels from Sham, PrI and PBBI were evaluated by Western blot at time points 4h, 24h, 3d and 7d post injury. Bands were measured and normalized to corresponding βactin levels. A representative blot at 7d post injury is shown in (A). Quantification corresponding to (B) tau-FL, (C) 40 kDa tau and (D) 22 kDa tau show significant loss of tau-FL and 40 kDa tau and significant increases in 22 kDa tau fragment through 7d. Statistical analysis of changes at each time point was conducted (K-W, * p<0.05, 2 way ANOVA # p<0.05; error bars: standard error of the mean, n = 8–10 per group).

**Fig 7:**
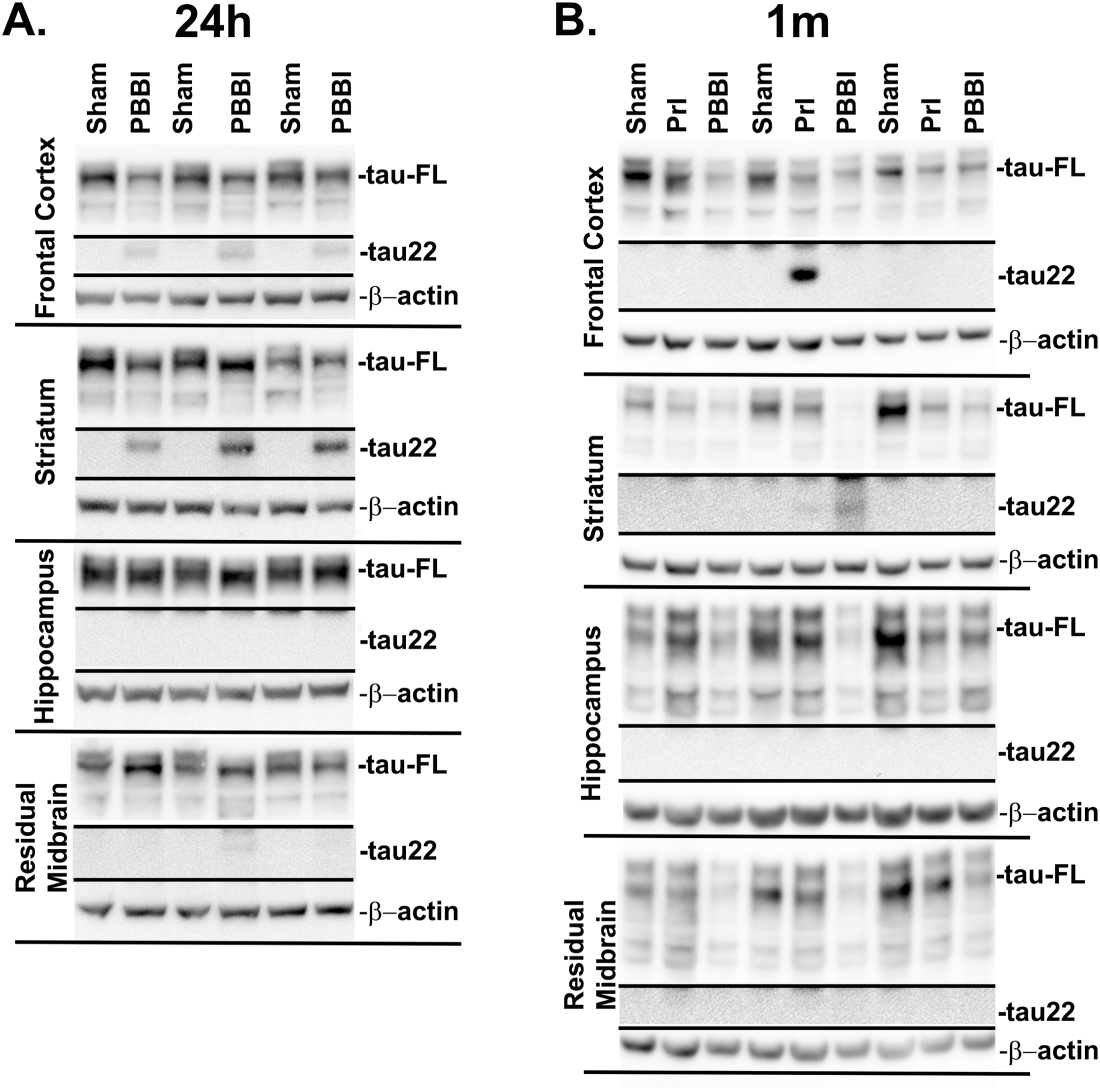
Pilot study: tau22 fragmentation in regions of interest. Tissue from specific regions of interest (Frontal Cortex, Striatum, Hippocampus, and Residual Midbrain) were qualitatively evaluated by Western blot for the presence or absence of tau22 fragmentation (A) 24h following PBBI (n = 3) and (B) 1m following PrI and PBBI (n = 3).

Following this analysis changes in tau-FL (55 kDa) and tau processing were evaluated following PrI and PBBI over four time points spanning the acute to sub-acute period post-injury (4h, 24h, 3d, and 7d). A representative blot shows sustained loss of tau-FL and the presence of a 22 kDa tau fragment (tau22) 7d post-injury (Fig 6A). Additional blots for all time points can be found in S8–S11 Figs. ROUT analysis identified 1 outlier from the PBBI tau-FL group, 2 outliers from the PrI tau-FL group at 3d, and 1 outlier from the PBBI tau-FL group at 7d, which were not included in final analysis. Analysis of quantification indicated that at 4h and 24h post-injury tau-FL was unaltered but was thereafter decreased substantially at 3d (PrI: -86%%; PBBI: -91%, K-W, * p<0.05, 2 way ANOVA # p<0.05), and 7d (PrI: -82%; PBBI: -92%, K-W, * p<0.05, 2 way ANOVA # p<0.05) (Fig 6B). A 40 kDa tau fragment showed significant decreases following PBBI in a non-linear pattern with initial decreases at 4h post-injury (-48%, K-W, * p<0.05) that partially resolved at 24h. However, at 3d and 7d levels were again decreased (3d: -90%; 7d: -89%, K-W, * p<0.05, 2 way ANOVA, # p<0.05). PrI injury showed more moderate changes with significant decreases at 3d and a decreased trend at 7d post-injury (3d: -67%, K-W, * p<0.05, 2 way ANOVA, # p<0.05; 7d: -64%, 2 way ANOVA, # p<0.05) (Fig 6C). In contrast, a 22 kDa tau fragment (tau22) increased following PBBI. ROUT analysis indicated 1 outlier in the PrI tau22 group at 7d which was not included in final analysis. Analysis of quantification showed significant post-PBBI increases at 4h (1620%, K-W, * p<0.05), 24h (2931%, K-W, * p<0.05, 2 way ANOVA, # p<0.05), 3d (2871%, K-W, * p<0.05, 2 way ANOVA, # p<0.05) and 7d (3653%, K-W, * p<0.05, 2 way ANOVA, # p<0.05). PrI induced a temporally similar pattern of increases at 4h (617%, K-W, * p<0.05), 24h (2248%, K-W, * p<0.05, 2 way ANOVA, # p<0.05) and 7d (1973%, K-W, * p<0.05, 2 way ANOVA, # p<0.05) but not at 3d (Fig 6D). Pilot studies qualitatively evaluating tau fragmentation in specific ROIs 24h post-PBBI indicated the presence of tau22 in multiple ROIs primarily in areas proximal to the injury tract (frontal cortex, striatum) rather than surrounding areas (hippocampus, residual midbrain) (Fig 7A). Pilot data shown in Fig 7A originated from studies already conducted in our primary model of penetrating injury PBBI. Although, PrI is sometimes used as control for injury induced by probe insertion alone but is not included in all studies as our studies and was not included in the 24h pilot study on ROIs. The pilot study in Fig 7B was included in order to discern whether or not penetrating TBI induced tau pathology in that were either within or distal from the injury core and this is captured with the more severe PBBI model. More comprehensive longitudinal studies of ROIs following PrI and PBBI are currently being conducted. Additional pilot studies qualitatively evaluating tau fragmentation 1m following PrI or PBBI injury detected tau22 in one third of both the PBBI and PrI animals. Thus, while in some animals tau22 is not present, in other animals PrI or PBBI clearly showed the presence of tau22 (Fig 7B).

### Characterization of TBI induced tau22 fragment

**Fig 8:**
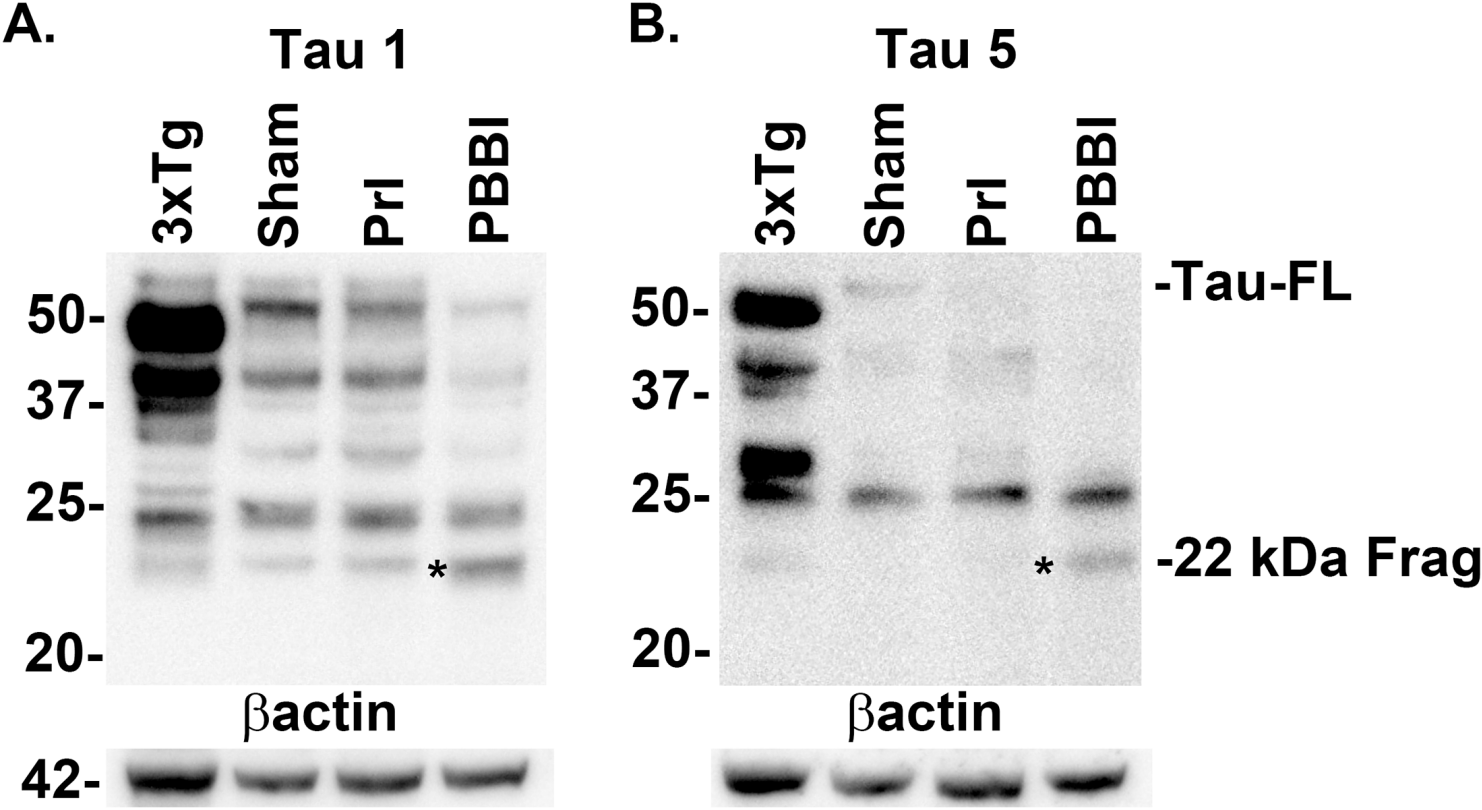
Epitope binding confirmed for Tau-FL and Tau22 using Tau1 and Tau5 antibodies. Specific binding to proline rich domain of tau confirmed for Tau-FL and tau22 fragment in samples 24h post-injury using (A) Tau1 and (B) Tau5 antibodies against total tau and compared to positive control transgenic AD model mouse brain lysate (3XTg-AD). The 22 kDa tau fragment (*) was detected by both total tau antibodies. Experiment replicated 3 times for (sham, PBBI).

**Fig 9:**
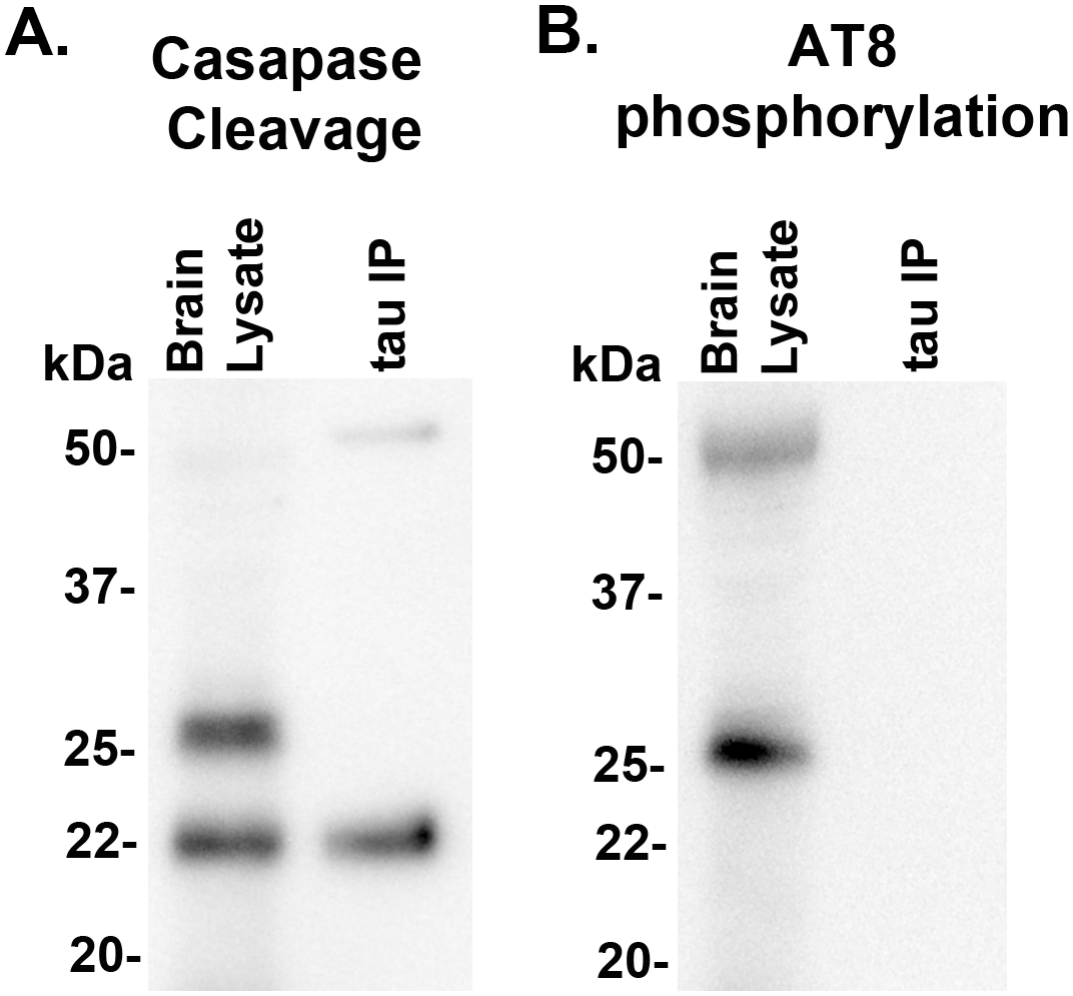
22 kDa Tau fragment binding confirmation. Protein levels from 7d post-injury PBBI brain lysate and 7d post-PBBI brain lysate that underwent immunoprecipitation with Tau 1 antibody (tau IP) were evaluated for evidence of (A) tau caspase cleavage at Asp421and (B) tau phosphorylation using AT8 antibody. Evidence of caspase cleavage of the 22 kDa tau fragment was seen using the tau Asp421 specific caspase antibody in both the original brain lysate as well as the Tau 1 immunoprecipitation product. Antibody AT8, specific for phosphorylation at sites S202, did not detect the 22 kDa tau fragment. Experiments replicated at least 3 times (sham, PBBI). AT8 phosphorylation experiments replicated twice (sham, PBBI).

To further characterize tau22 specificity to tau-FL, the recognition of this injury induced fragment was analyzed using additional tau antibodies (Tau 1 and Tau 5) which map to an area of the proline rich region of tau adjacent to the 5E2 antibody used previously. Both Tau 1 and Tau 5 antibodies showed strong tau22 recognition in both the positive control (3xTg-AD mouse brain lysate) as well as PBBI tissue (Fig 8A and 8B, S12 Fig). In addition, the possibility that the tau22 fragment may be the result of caspase cleavage was investigated. First the tau and tau cleavage products were immunoprecipitated using the Tau 1 antibody. Following this both the original PBBI brain lysate and the immunoprecipitation product were evaluated with a tau antibody specific for the epitope exposed following caspase enzymatic cleavage at aspartic acid 421 (Asp421). In both the original PBBI lysate and the tau specific immunoprecipitation product the caspase cleavage specific antibody recognized tau22 (Fig 9A, S13A Fig). The caspase cleavage antibody was also mapped (Fig 2) and taken together with binding of Tau1 antibody suggest tau22 includes segments of the proline rich domain and the repeated binding domains of the tau protein. Finally, tau22 was evaluated to determine whether the fragment was phosphorylated at serine 202 using the AT8 tau antibody. Neither PBBI brain lysate nor the tau specific immunoprecipitation product showed serine 202 phosphorylation of tau22 (Representative blot: Fig 9B, additional blot: S13B Fig).

## Discussion

This study is the first to measure TBI induced changes in APP CTFs, Aβ40 and Aβ40 levels in the non-transgenic rat and shows that APP CTFs, Aβ40, Aβ42 are detectable and quantifiable in the absence of human familial mutations or over-expression systems. This allows for the study of APP and tau processing in a variety of TBI rat models, including our established PBBI model. Furthermore, our goal was to also investigate how changes in these AD related processes occur in the acute and sub-acute periods following severe penetrating TBI given that the study of such processes thus far has been limited to less severe models of TBI. Our analysis of the acute through sub-acute period post-injury time frame showed that PBBI produced significant decreases in the levels of intact APP by 3d post-injury that which were sustained through 7d post-injury but were not evident following PrI alone. In contrast, moderate TBI have either failed to produce any significant changes in APP-FL [23, 24] or produced effects limited to an acute (24h) increase in APP-FL [17]. As APP has normal function both in synaptic structure and neurite outgrowth [33, 34], the dramatic and sustained loss of this protein following PBBI may have long-term adverse effects on the recovery of neuronal signaling following severe TBI. In accordance with the observed reductions in APP-FL, significant increases in APP βCTF levels were detected. This is indicative of increased pathogenic βsecretase cleavage. The observed increases in APP βCTF sustained throughout the acute to sub-acute time period post-PBBI, reaching peak expression at 24h post-injury. However levels remained 9-fold higher than control levels at 7d post-injury. In contrast, in models of moderate TBI APP neither βCTF nor Aβ levels are significantly upregulated past 3d [23, 24]. APP βCTF upregulation following PrI closely mirrored what was observed following PBBI, particularly at 7d post-injury where they were almost 7-fold higher than sham levels.

To confirm that the sustained increases in β-cleavage in the sub-acute period corresponded to increased Aβ, Aβ40 and Aβ42 levels were directly analyzed at 7d post-injury. Although measuring non-transgenic rodent Aβ forms was difficult due to low protein abundance and limited assay availability, analysis here clearly showed increases in both these Aβ forms at 7d post-injury. In contrast, moderate TBI studies led to acute increases in Aβ40 and Aβ42, but these changes were resolved by 7d [23, 24]. In this study direct analysis of amyloid was restricted to 7days to confirm our interpretation of more temporally complete analysis of APP β-cleavage indicated by βCTFs. Future studies will evaluate how much further these changes may be detected post-injury and confirm the continued presence of Aβ at more chronic time points. Changes in Aβ were increased with PBBI inducing more Aβ40 and Aβ42 than PrI. This observed relationship between injury severity and the amplitude of Aβ40 and Aβ42 increases in the rat may correspond to what has been shown in human studies of World War II veterans where the severity of head-injury sustained in early adulthood during active duty was correlated with increased risk of late-onset sporadic AD [12].

Interestingly, both PrI and PBBI lead to an acute spike in αCTF levels. A possible explanation for this is that loss of APP-FL acutely leads to homeostatic mechanisms increasing APP production in order to maintain steady state APP levels. The result may be a temporary increase in the non-pathogenic αCTF as part of APP ongoing APP and α-secretase cleavage. Following probe injury a similar spike in αCTF was seen subacutely, suggesting that in this lower severity penetrating injury homeostatic mechanisms are activated while in the more severe PBBI the ability to maintain APP-FL levels is lost and thus homeostatic mechanisms are only able to maintain αCTF at levels similar to control rather than induce an αCTF spike. The α-secretase cleavage of APP generates both αCTFs, measured here, as well as the corresponding alpha secreted APP (sAPPα), a peptide known to regulate neural progenitor cell proliferation [35–37]. Thus fluctuating αCTFs and potentially the corresponding sAPPα may affect innate neurorestorative processes following severe TBI.

Concurrent with demonstrated increases in pathogenic Aβ production and concomitant reductions in neurorestorative APP α-cleavage products, significant reductions of tau-FL and increased levels of a potentially pathogenic tau22 cleavage product were detected post-TBI. Loss of tau-FL occured following either PrI or PBBI and 3d and 7d post-injury where only trace amounts of intact tau remained. The microtubule-associated tau protein is expressed primarily in axons, promotes microtubule stability and neurite outgrowth. In addition, it facilitates enzyme anchoring and the anterograde and retrograde axonal transport of organelles [38]. Thus the loss of intact tau protein may be indicative of loss of axonal integrity and impaired neuronal function even within surviving neurons following TBI. This feature is likely, since the PBBI model is characterized by both pathologies and includes significant portions of striatal white matter in the injury core and peri-lesional zones [27]. Ultimately, the loss of tau in axonal tracts would lead to neuronal damage at the soma. In parallel to the effects seen with the 55kDa tau-FL protein, a 40 kDa tau protein showed progressive decreases following PBBI starting by 4h. Again, both PrI and PBBI showed dramatic decreases at 3 and 7d post-injury, suggesting that this is another isoform of tau lost following tissue damage rather than a tau cleavage product. Although rat tau isoforms have yet to be fully sequenced and characterized, this 40 kDa tau form may be similar to human tau isoform 4, which is of comparable size (National Center for Biotechnology Information, NP_058525.1). Thus, following severe TBI multiple forms of tau are lost which may result in increased axonal instability.

In contrast to the loss of tau isoforms, both PrI and PBBI led to dramatic increases in a tau22 fragment. Overall, PBBI led to greater and more constant increases in tau22 in comparison to PrI where levels were reduced at 3d only to rebound at 7d. Early results indicate tau22 fragmentation occurs primarily in areas in close proximity to the injury tract (frontal cortex, striatum) and that in a limited number of animals remains present up to 1m post injury. Of note, both PrI and PBBI showed the presence of tau22 at 1m. PrI is of significantly lower injury severity than PBBI since there is no balloon inflation and temporary cavity in the injury formation. However, it is still a penetrating injury where a steel probe is inserted at substantial depth into the brain as exemplified in Fig 1C. At 7d post-injury tau22 levels with PrI were lower than PBBI levels but were still significantly upregulated in relation to shams. By 1 month, many animals may have cleared away tau22, possibly by microglial phagocytosis or other mechanisms. However, given that at 7d both PrI and PBBI showed extensive levels of tau22 it is not that surprising that in both PrI and PBBI some animals still show detectible levels of tau22 in regions close to the probe insertion tract (frontal cortex and striatum) at 1 month post-injury. The strong presence of tau22 in any animals shows that the continued presence of this protein through 1m is possible and thus indicates increased risk for the continuation of this pathology long-term, possibly setting the stage for more chronic neurodegenerative process to occur in a subset of animals. More comprehensive chronic studies will further elucidate this risk. Other preclinical studies have also shown that TBI leads to increased tau truncation [18, 19]. Studies of AD brains report that 22 kDa tau fragments have been found to be increased in the neuronal synapses of AD subjects, that these tau fragments correlate with mitochondrial dysfunction, and have been proposed as CSF biomarkers with low specificity but high sensitivity for a variety of tauopathies including AD, frontotemporal lobar degeneration, Parkinson’s disease with dementia, and vascular dementia [3, 4, 39]. However, a variety of other truncated tau forms have also been implicated in AD progression [5, 40, 41]. In addition, tau truncation fragments can induce the formation of novel *tau* fragments in a self-perpetuating manner and are found primarily in the misfolded fraction of *tau* and can lead to the acceleration of neurofibrillary pathology [5]. Certainly the degree of tau22 accumulation by 7d post-injury (20-fold or higher) supports the theory that a self-perpetuation process may lead to these drastic increases in tau truncation and loss of full length tau isoforms. This study demonstrated that penetrating TBI leads to a similarly sized *tau* fragment as that seen in studies by Amadoro et al. (2010) and, to confirm the specificity of this tau22 fragment, showed that antibodies recognizing three separate but adjacent epitopes of *tau* were able to recognize tau22, indicating that this fragment contains portions of the proline rich domain of tau. Our results indicate that this tau22 fragment is the result of caspase cleavage at the C-terminal end and the resulting epitope mapping indicates tau22 spans the proline rich domains and the microtubule binding domains and is cleaved at the C-terminal end rather than the an N-terminal cleavage fragment studied by Amadoro et al. (2010). Tau22 did not show phosphorylation at the S202 site. Analysis of other tau phosphorylation sites was limited by the lack of commercially available high affinity antibodies for western blot analysis. Thus, additional studies are necessary to fully examine the phosphorylation state of tau22 as tau phosphorylation has long been linked to in the development of AD tangles [42, 43]. However, there is clear evidence that C-terminal caspase cleavage of tau can also act as an inducer of tau aggregation in AD and has been associated with both early and late-stage neurofibrillary tangles [5, 40, 41]. Sources of tau caspase cleavage could be pro-apoptotic mechanisms within neurons following injury as well as post-phagocytosis mechanisms within microglia or infiltrating macrophages following blood brain barrier disruption. While follow-on studies would be necessary to directly determine whether this tau cleavage leads to tau aggregation, these finding indicate that severe TBI leads to both an overwhelming loss of structurally critical tau isoforms as well as a dramatic accumulation in the potentially pathogenic tau22 fragment.

## Conclusions

Traumatic brain injury (TBI) is an established a risk factor for the later development of Alzheimer’s disease (AD). As AD driven neuropathology does not manifest until the later stages of life in the human and is not yet known to occur in non-transgenic rodents, this is a mechanistic study of the effects of severe TBI on APP and tau processing and this model is not expected to replicate AD. We are not attempting to show AD pathology explicitly, but to study possible underlying mechanisms that may lead to later pathology, either AD, or other neuropathologies. Penetrating TBI with increasing severity was evaluated in rats, and showed rapid and substantial elevation in tau fragment accumulation similar to that seen in human AD brains. In addition, APP β C-terminal fragments and Aβ peptides showed sustained increases that persisted one week post- injury. Future studies may further elucidate the regional distribution and how long post-injury these changes are sustained. This is the first data to show these AD-relevant changes in non-transgenic rats, specifically in a model of severe TBI. This study may more accurately parallel TBI-induced molecular changes related to sporadic AD than models incorporating AD familial mutation over-expression systems. The findings indicate both APP and tau cleavage was dramatically altered throughout acute and subacute periods post-injury. Importantly, classical animal models of AD, such as transgenics harboring AD-causing mutations take months to a year to show AD pathology such as plaques and tangles. As such, the highly significant changes in amyloid processing and tau cleavage seen here through one week indicate more extensive and prolonged TBI pathology compared to other similar studies to date. The time frame studied here is relatively early in comparison to the incubation period involved in the formation of AD neuropathology such as plaques or tangles. Yet these acute and subacute outcomes may set the stage to fully elucidate molecular mechanisms that begin to define how moderate and severe TBI may contribute to the development of AD or other tauopathies at much later times, particularly in the absence of familial mutations. In regards to the development of treatments for TBI, the observed alterations in APP processing and tau truncation may represent useful preclinical targets to evaluate putative therapeutics following PBBI. One possible candidate may be the LXR agonist T090317 which in moderate TBI was able to reduce the TBI induced Aβ response [24] while estrogen treatment has been proposed to reduce the caspase-induced tau truncation [44]. Further studies will determine if these changes are perpetuated at chronic time-points post-TBI and the extent to which the tau22 fragment can be detected in biofluids as a potential biomarker of disease progression or be used as a theranostic for evaluating treatment efficacy.

## Supporting Information

S1 Fig Western blots of APP 4h post-injury (All blots). (TIF)

S2 Fig Western blots of APP 24h post-injury (All blots). (TIF)

S3 Fig Western blots of APP 3d post-injury (All blots). (TIF)

S4 Fig Western blots of APP 7d post-injury (All blots). (TIF)

S5 Fig β-actin levels at 4h, 24h, 3d and 7d post-injury. (TIF)

S6 Fig Aβ40 Chemiluminescent ELISA 7d post-injury. (TIF)

S7 Fig Aβ42 Chemiluminescent ELISA 7d post-injury. (TIF)

S8 Fig Western blots of tau 4h post-injury (All blots). (TIF)

S9 Fig Western blots of tau 24h post-injury (All blots). (TIF)

S10 Fig Western blots of tau 3d post-injury (All blots). (TIF)

S11 Fig Western blots of tau 7d post-injury (All blots). (TIF)

S12 Fig Tau22 binding using Tau1 and Tau5 antibodies: Additional replicates of western blots. (TIF)

S13 Fig Additional replicates: Tau caspase cleavage, tau phosphorylation: Additional replicates of western blots. (TIF)
