# Supplementary figures and images for: Subacute Changes in Cleavage Processing of Amyloid Precursor Protein and Tau following Penetrating Traumatic Brain Injury
Source: PLoS One. 2016 Jul 18;11(7):e0158576. doi: 10.1371/journal.pone.0158576 (PMC4948774; doi:10.1371/journal.pone.0158576)

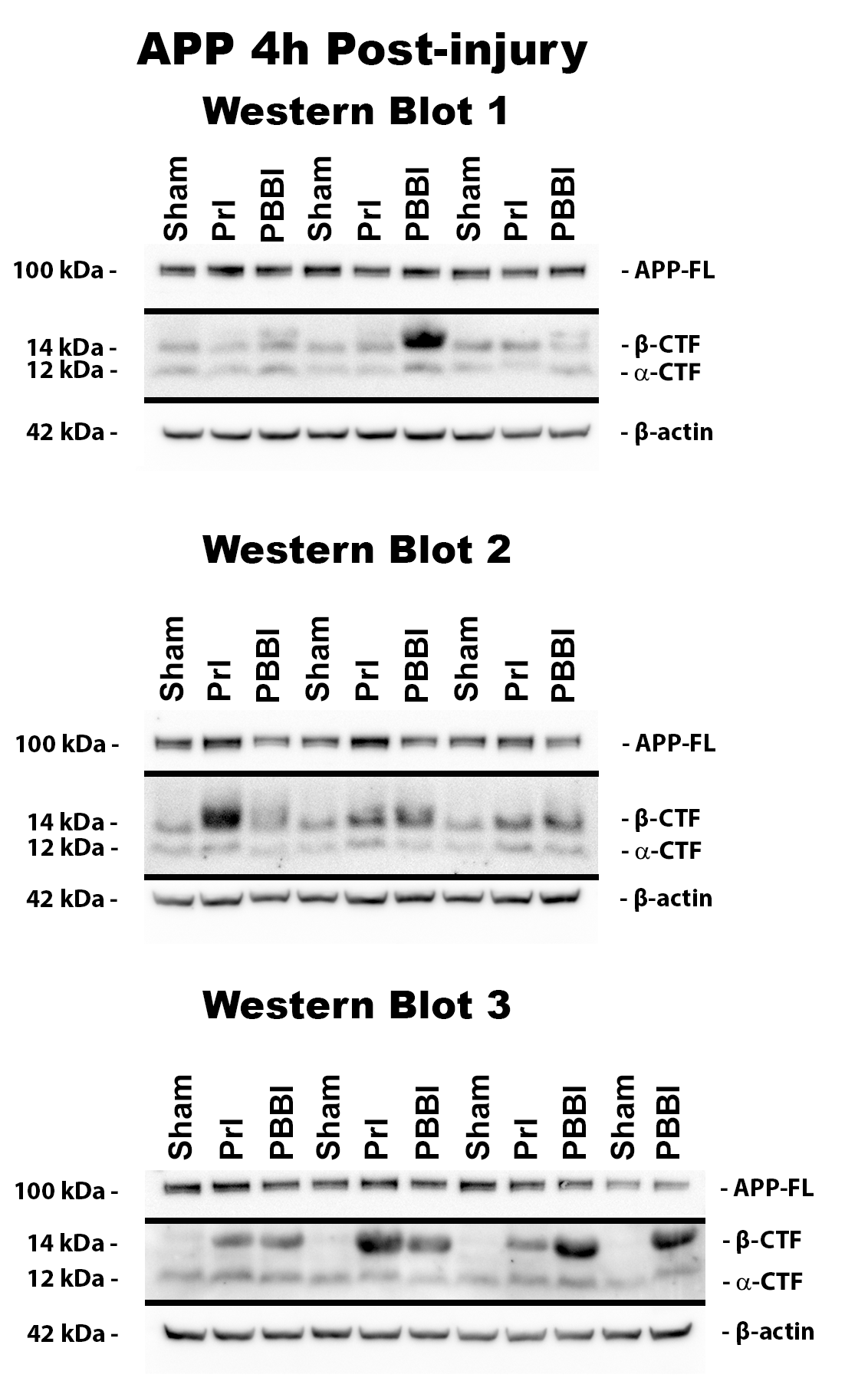

Supplement: S1 Fig — (TIF) [file pone.0158576.s001.tif]

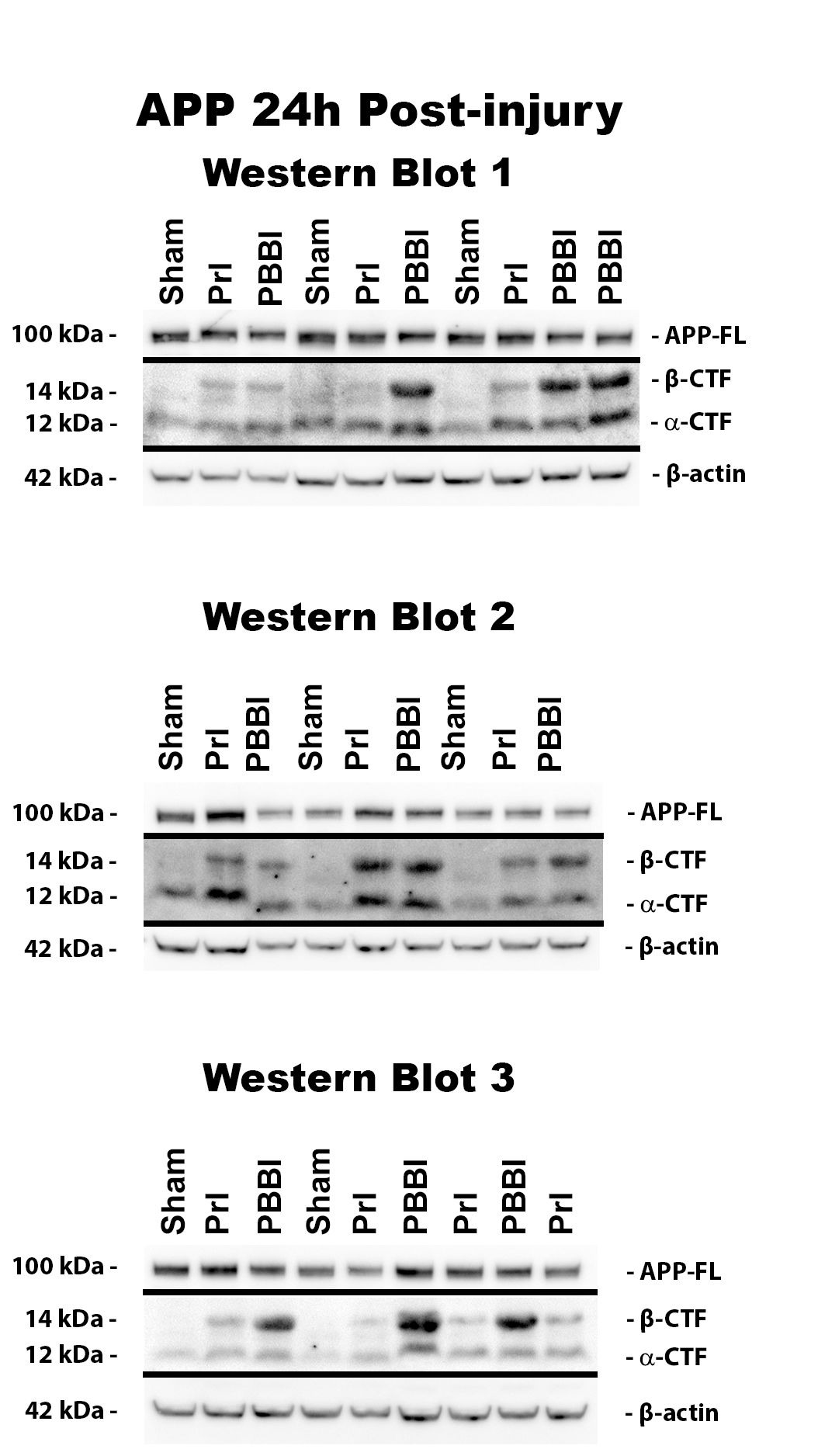

Supplement: S2 Fig — (TIF) [file pone.0158576.s002.tif]

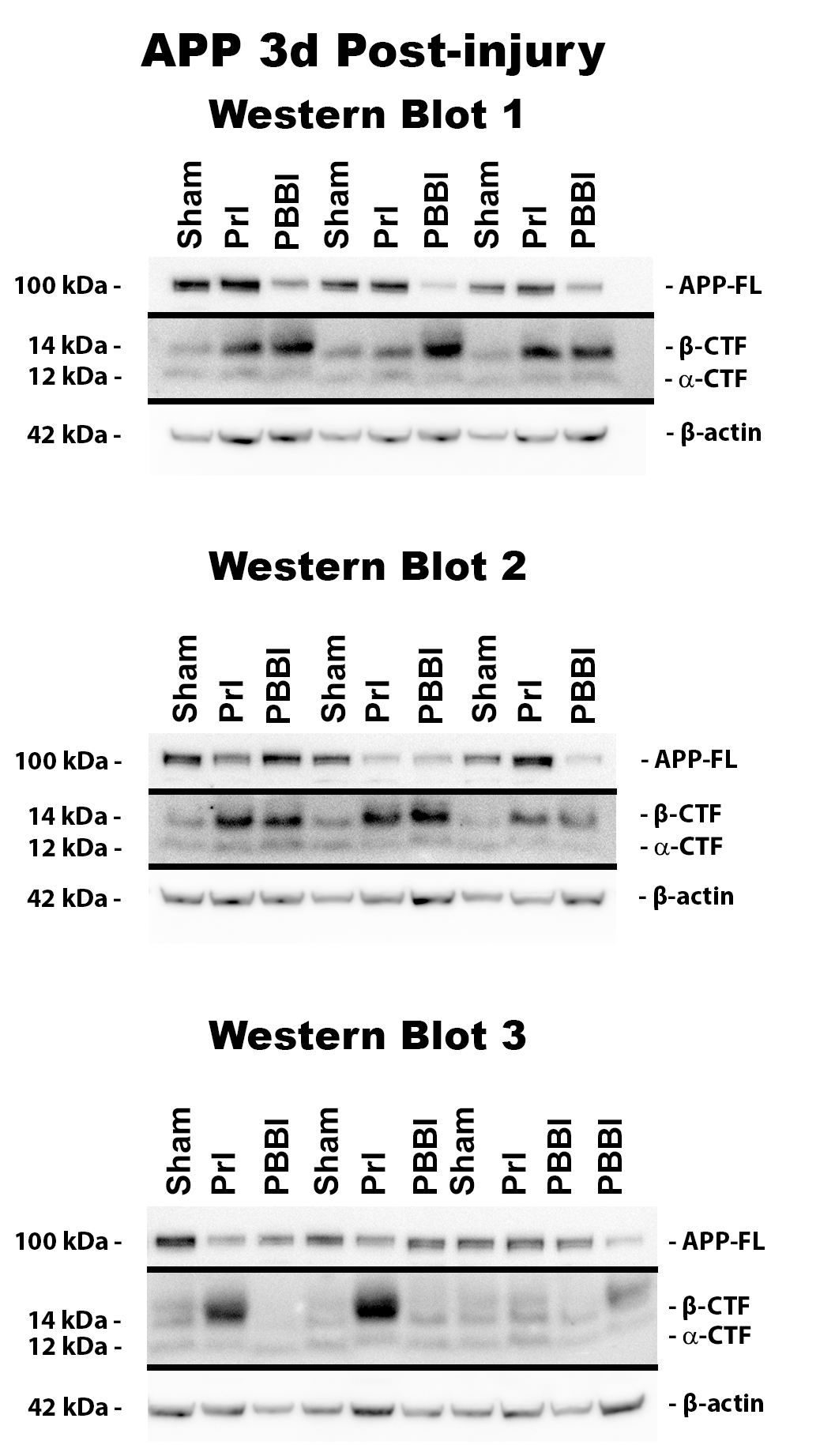

Supplement: S3 Fig — (TIF) [file pone.0158576.s003.tif]

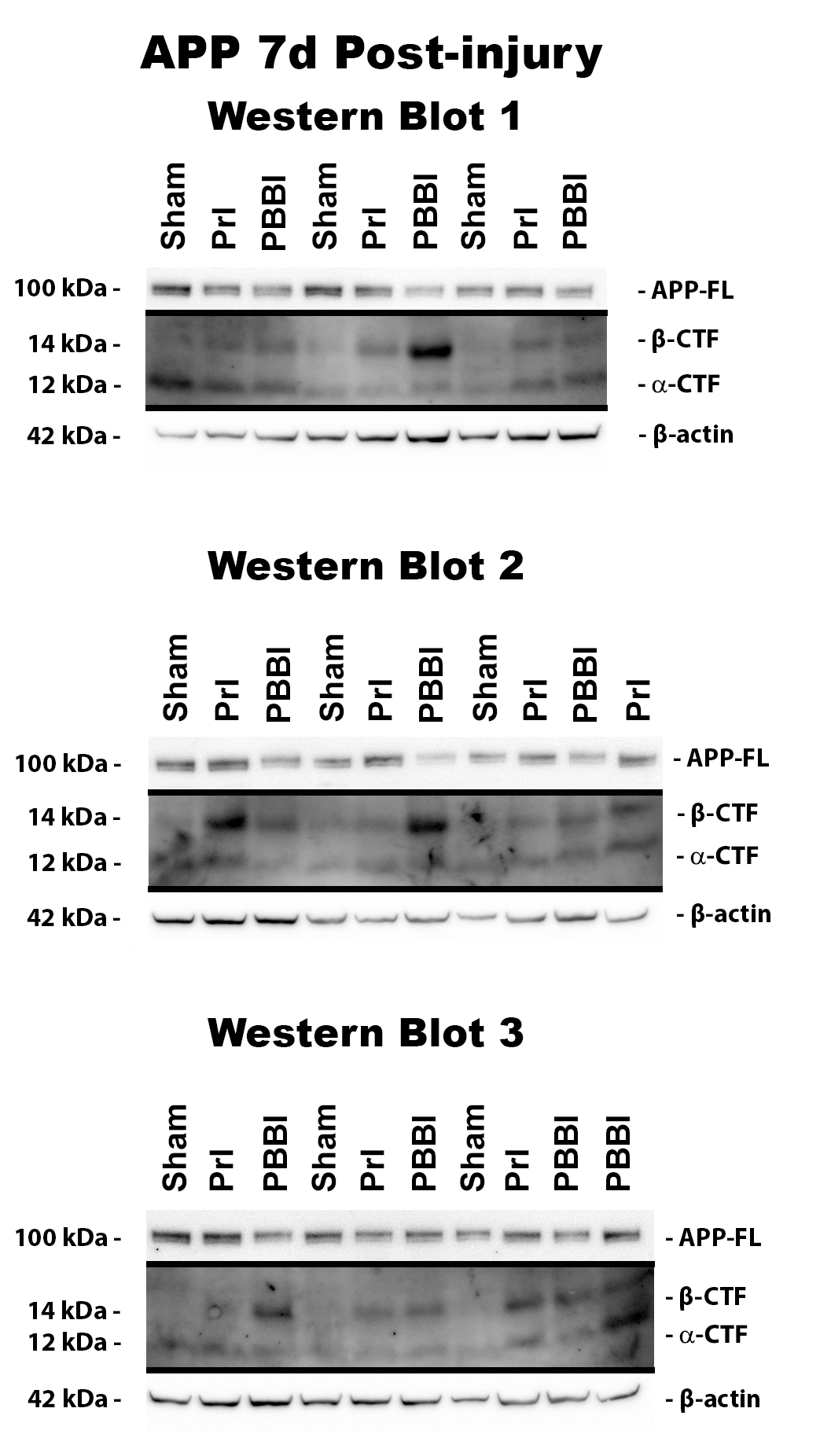

Supplement: S4 Fig — (TIF) [file pone.0158576.s004.tif]

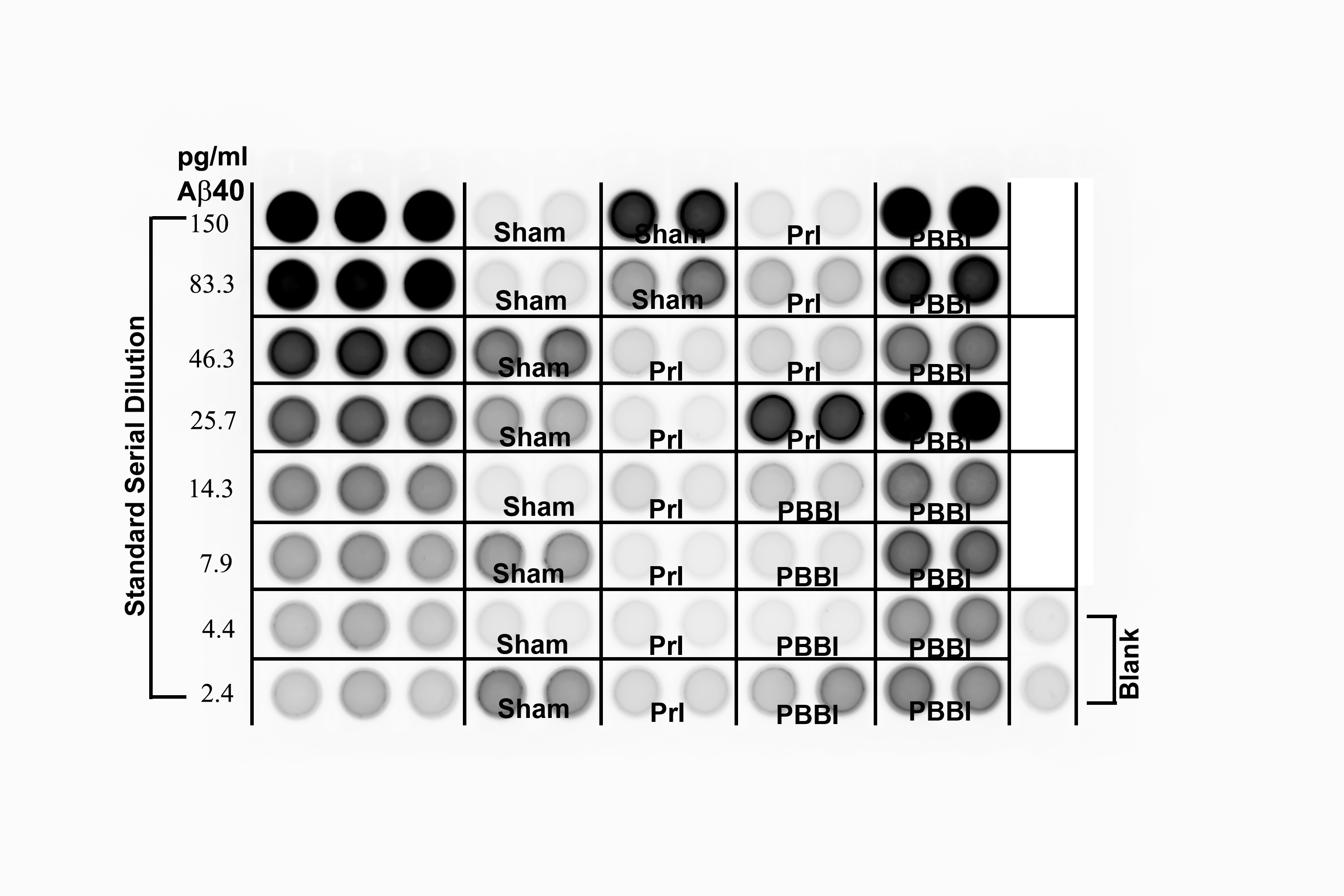

Supplement: S6 Fig — (TIF) [file pone.0158576.s006.tif]

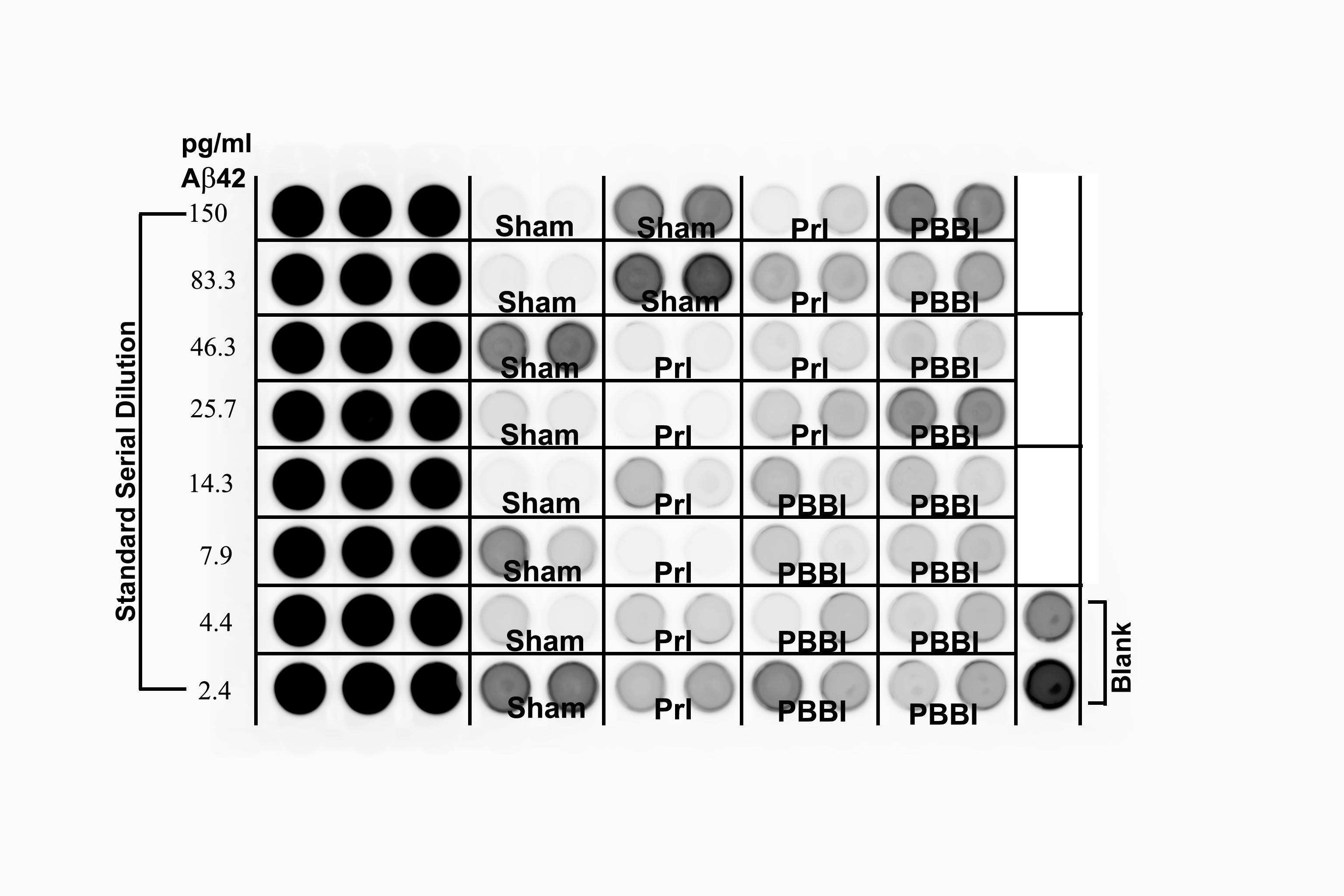

Supplement: S7 Fig — (TIF) [file pone.0158576.s007.tif]

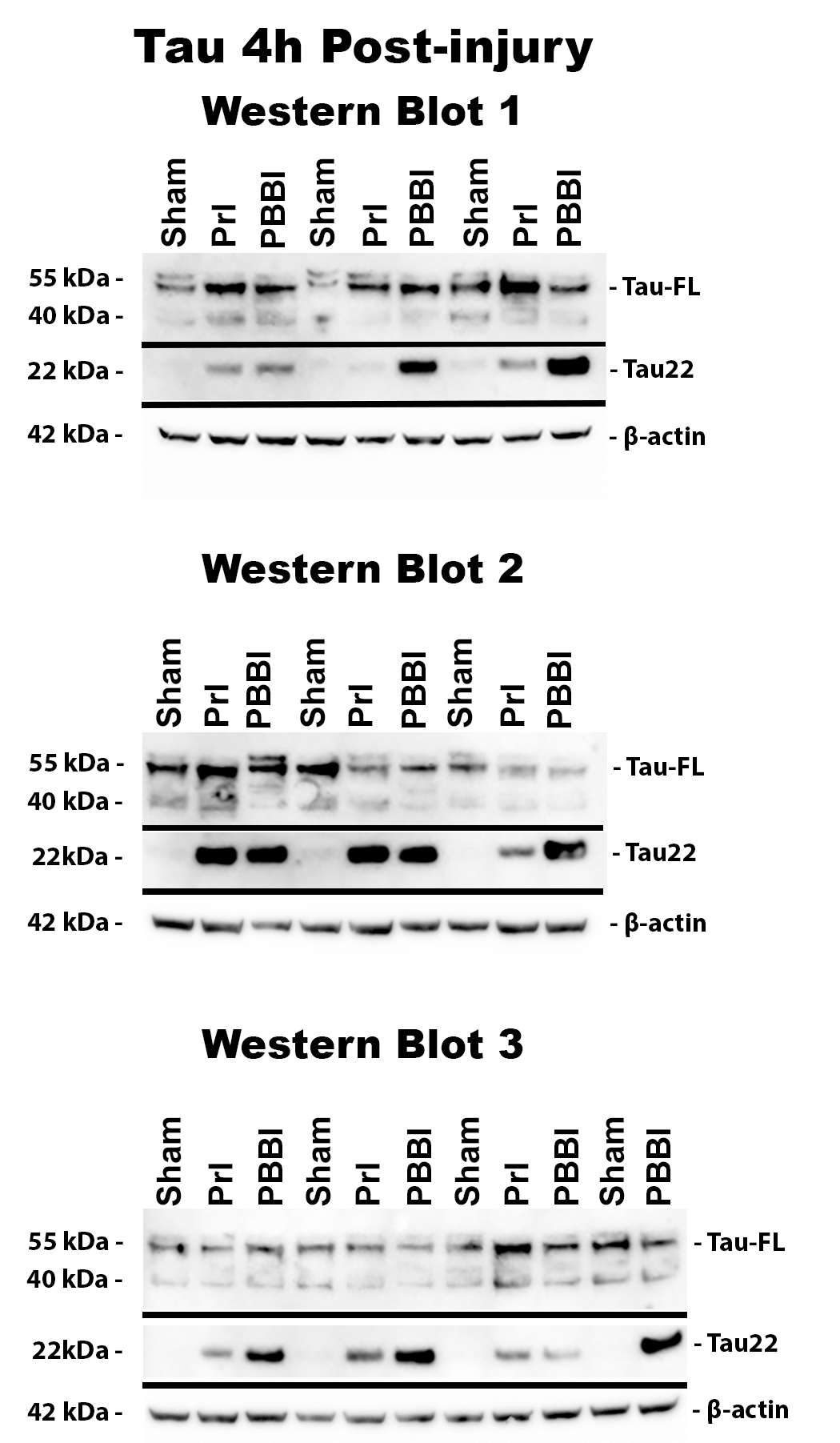

Supplement: S8 Fig — (TIF) [file pone.0158576.s008.tif]

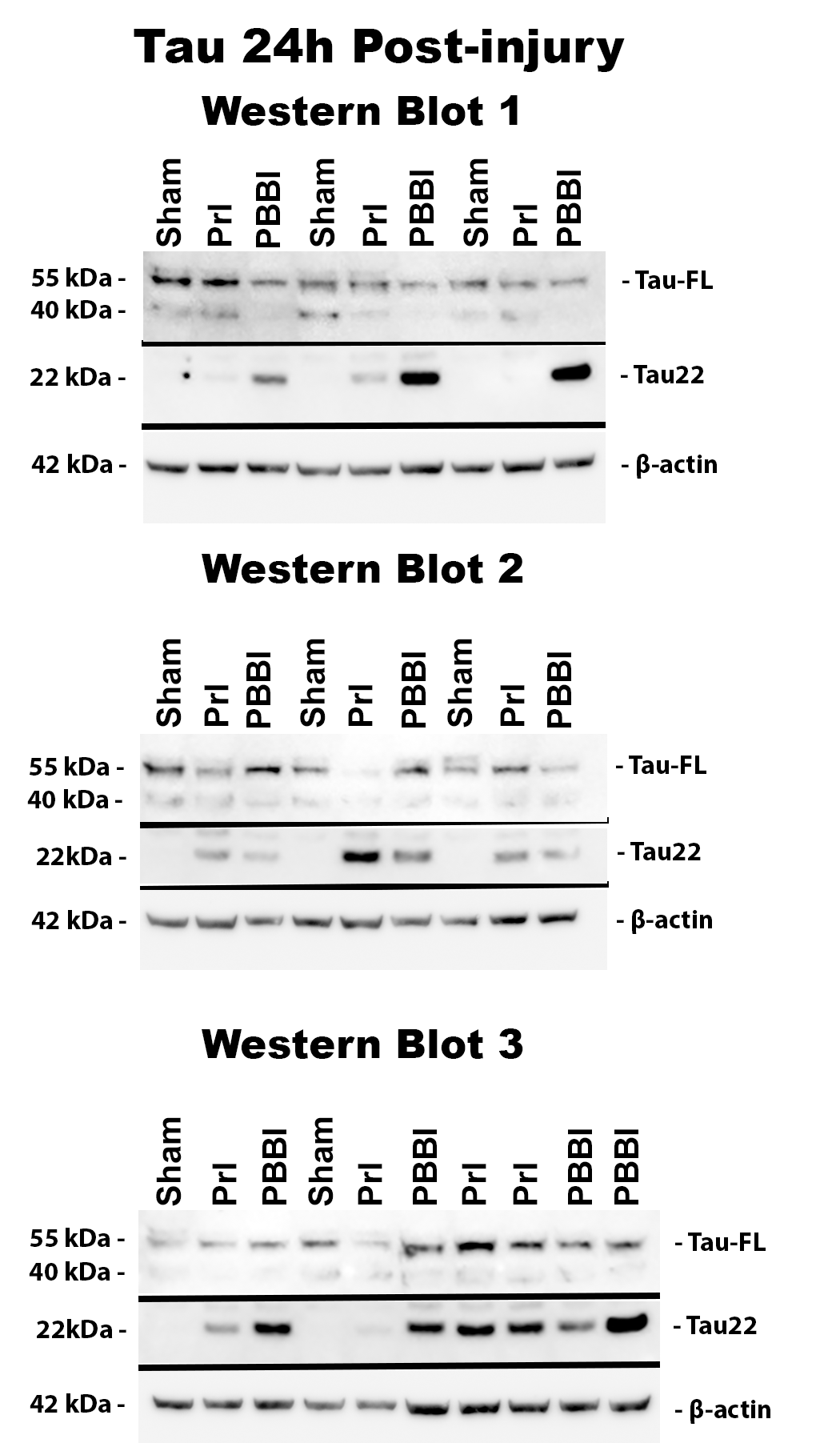

Supplement: S9 Fig — (TIF) [file pone.0158576.s009.tif]

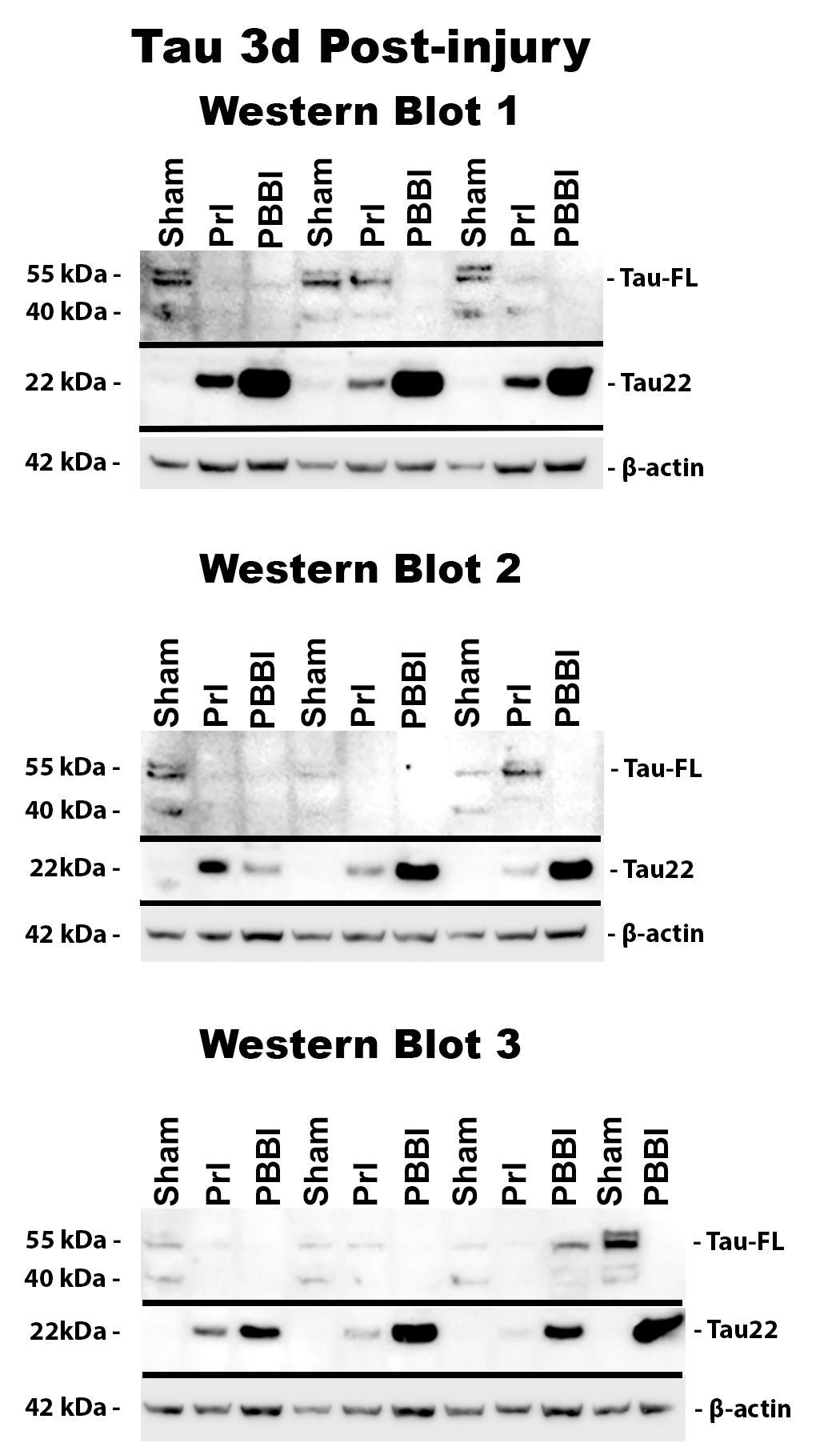

Supplement: S10 Fig — (TIF) [file pone.0158576.s010.tif]

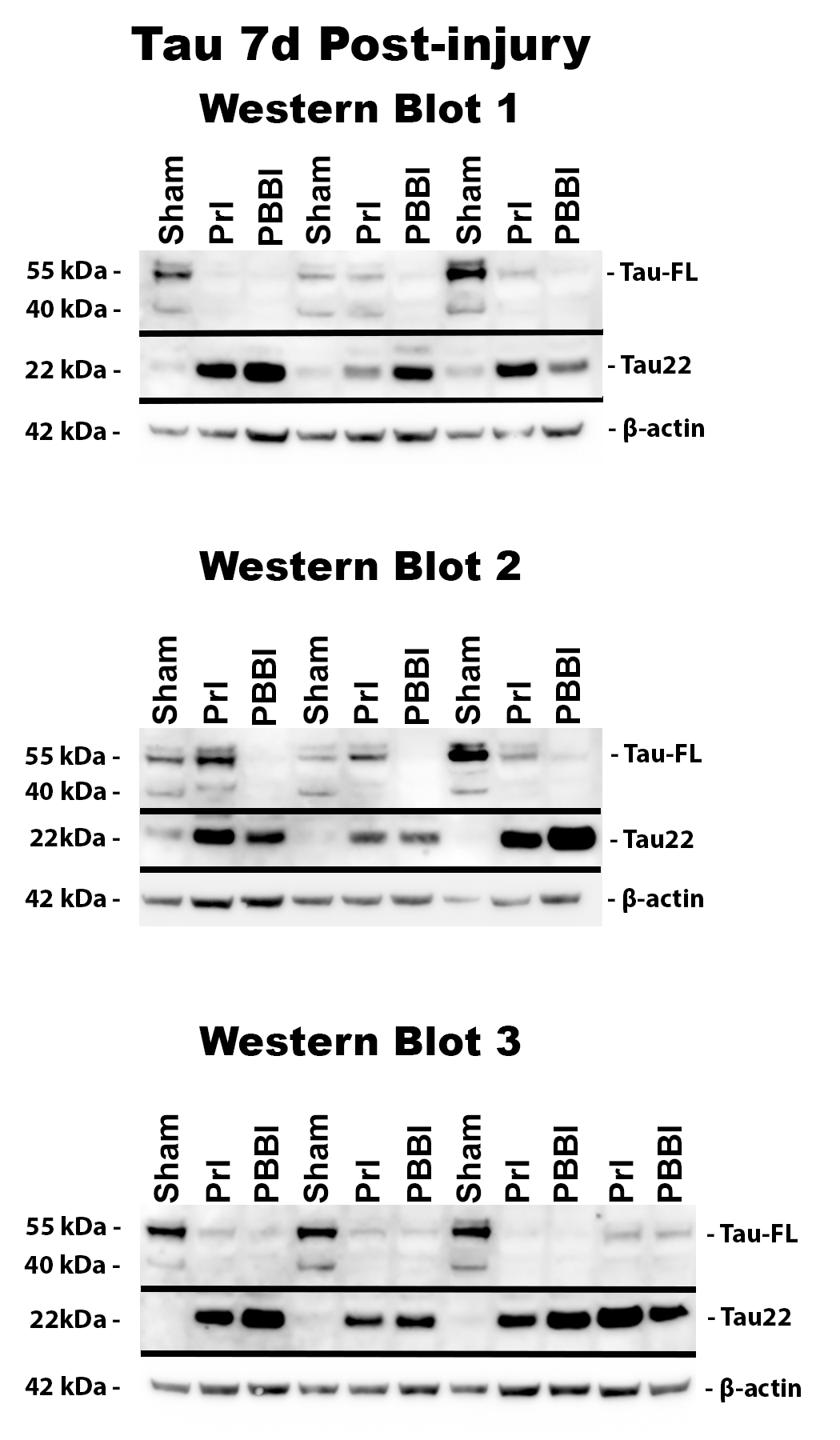

Supplement: S11 Fig — (TIF) [file pone.0158576.s011.tif]

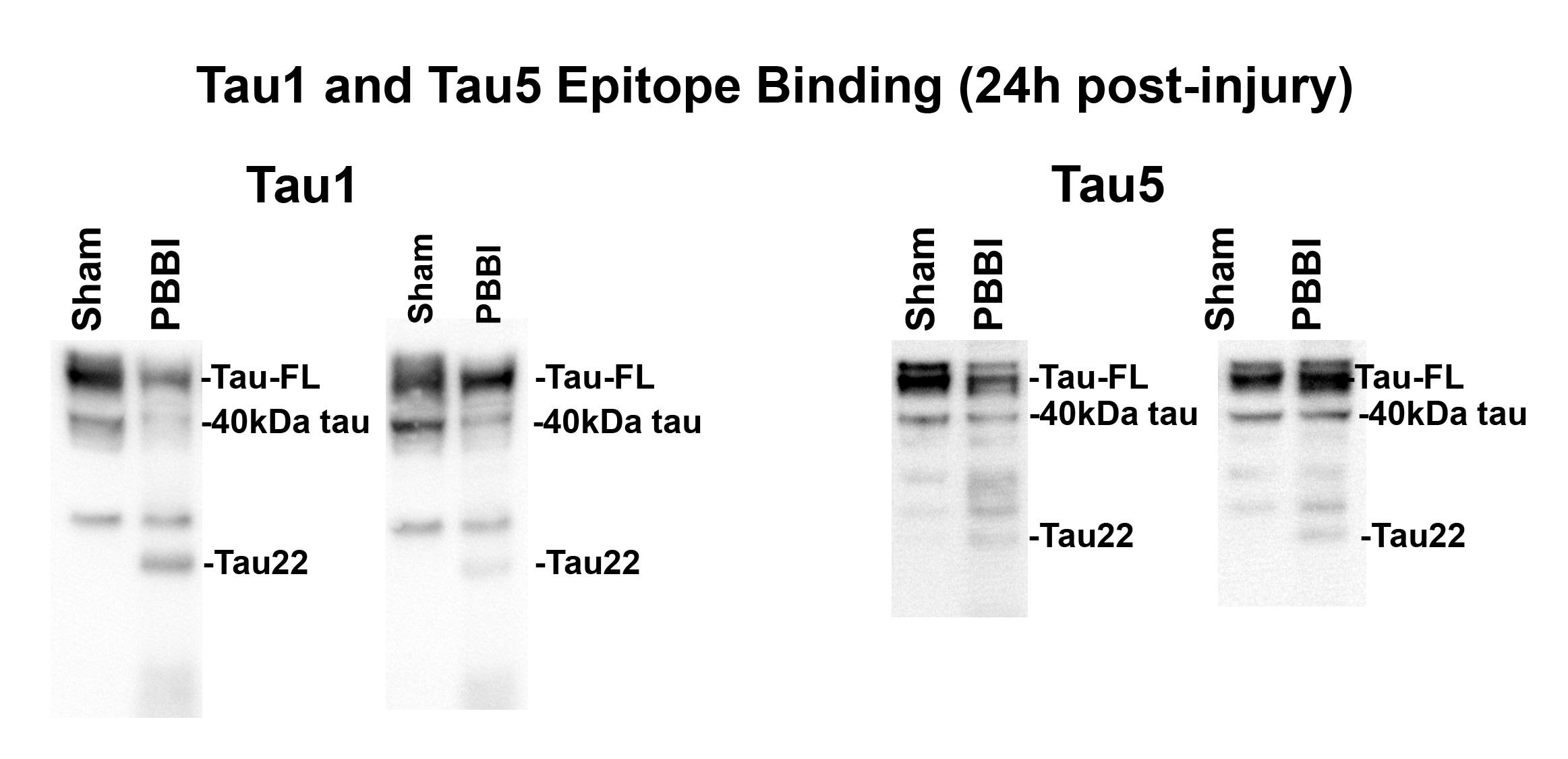

Supplement: S12 Fig — (TIF) [file pone.0158576.s012.tif]

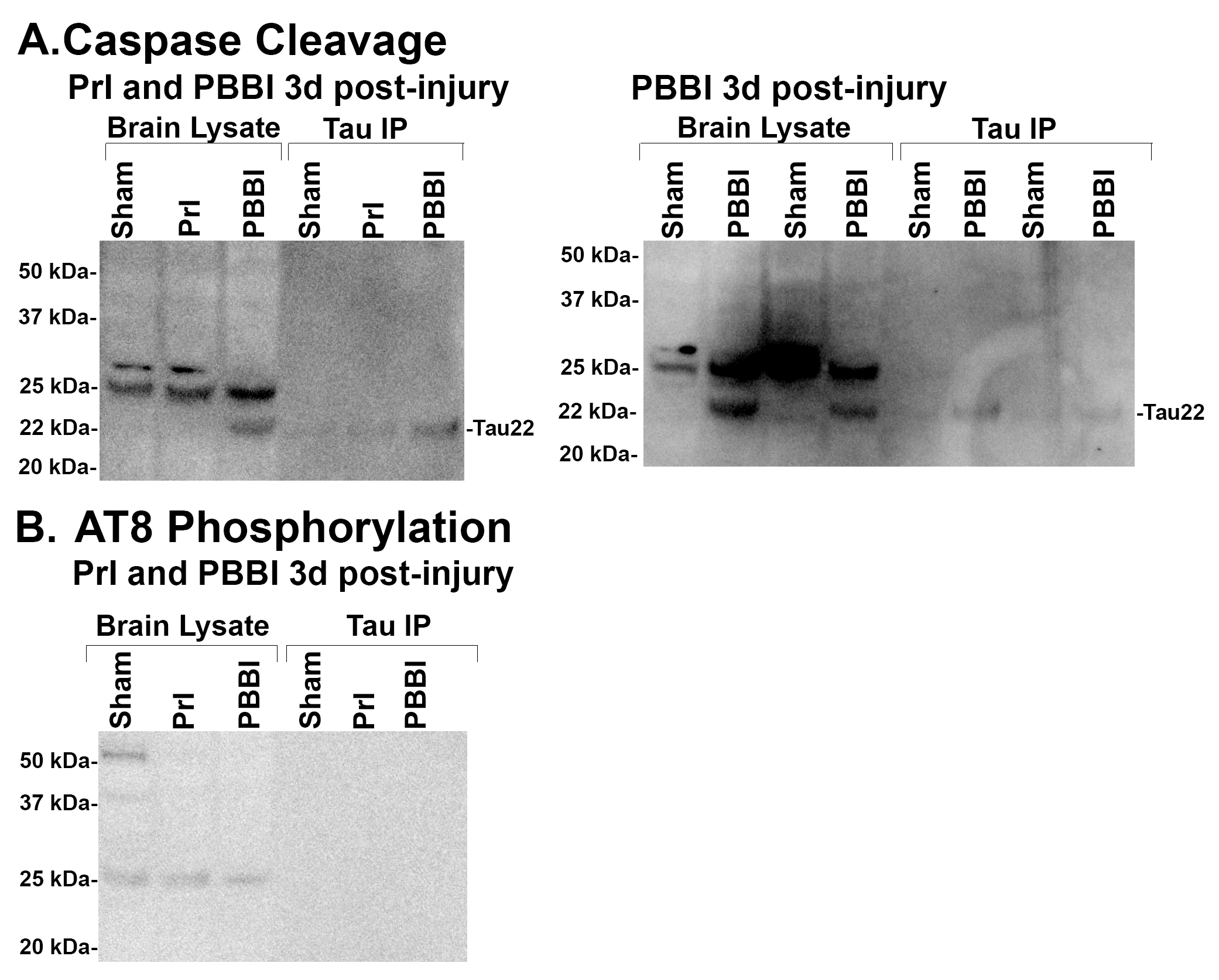

Supplement: S13 Fig — (TIF) [file pone.0158576.s013.tif]
